# The use of social media in social care: a systematic review of the argument-based ethics literature

**DOI:** 10.1007/s11019-025-10269-4

**Published:** 2025-05-03

**Authors:** Tijs Vandemeulebroucke, Larissa Bolte

**Affiliations:** Bonn Sustainable AI Lab, Institut für Wissenschaft und Ethik, Universität Bonn, Bonner Talweg 57, 53113 Bonn, Germany

**Keywords:** Social care, Social media, Ethics, Social work, Therapy

## Abstract

Digital technologies, especially social media, have become everyday tools. In care settings, the use of social media is considered a possible guarantee to maintain quality practices. This trend is specifically relevant for social care, including social work, psychology, psychiatry, rehabilitation etc., due to their communicative nature. Nevertheless, this use is joined by ethical vulnerabilities. To get insight into these, a systematic review of relevant normative-ethical literature was carried out following a 4-step methodology: developing ethical-conceptual questions; a literature search in four electronic databases (CINAHL, Philosopher’s Index, Web of Science, ProQuest Database Psychology); assessment and inclusion of articles based on predefined criteria; extracting, analysing, and synthesizing reported data. Thirty-three articles were included, showing that current ethical debates are governed by nine themes: Benefits of social media; Relations, limits, and boundaries; Searches; Privacy, confidentiality, and trust; Documentation and records; Competency and client suitability; Consultation and referral; Informed consent; and Identity and image. We found that most ethical literature on social media use in social care settings adheres to the principles of biomedical ethics (respect for autonomy, beneficence, non-maleficence, justice) and to an ethics of carefulness, i.e. an ethics which takes social media for granted and considers its impact only on the particular therapeutic relationship. It loses sight of those ethical issues which occur on organizational, societal, and global levels. A full account of the ethics of social media use can only be given by considering these different levels and by informing the ethics of carefulness by an ethics of desirability.

**Supplementary Information:**

The online version contains supplementary material available at 10.1007/s11019-025-10269-4.

## Introduction

In all dimensions of human life, digital technologies have entered and made their presence felt. Especially those technologies that came to be known as ‘social media’ seem to be commonplace in many people’s lives. In 2024, it is projected that 62.2% of the global population will be involved with social media in one way or another, with major differences between the global north and the global south (We are Social, DataReportal and Meltwater 2024). Looking closer to the authors’ home, it is projected that in Germany almost 79.9% of the population will use social media at least once per month in 2024 (Dixon 2022).

Social media can be characterized as those information and communication technologies that are used to establish digital social interaction. These technologies cover social networking sites (e.g., Facebook, LinkedIn), blogs (e.g., Tumblr), microblogs (e.g., X (formerly Twitter)), and text-, calling-, photo-, or video message platforms (e.g., Instagram, Whatsapp, Snapchat, TikTok). Social media create media networks that form the basis of digital relations, transforming social media users into active information producers by nudging them into sharing information (Fang et al. 2014). These networks facilitate synchronic interaction, in which the interaction between the different parties occurs in real-time (e.g., calling, video-calling), and asynchronic interaction, in which the moments of interaction between the different parties are spread over time (e.g., messaging, postings).

Because social media are so deeply integrated into people’s lives, it should not be surprising that healthcare and supporting services are exploring their potentialities to provide certain services. Especially in social care practices, the relevance of these technologies cannot be discounted (Chan 2016; Rosenberg et al. 2021; Westwood 2019). Here, we rely on a broad conception of social care inspired by care ethics and especially Bernice Fisher’s and Joan Tronto’s characterization of care as “[…] a species activity that includes everything that we do to maintain, continue and repair our ‘world’ so that we can live in it as well as possible. That world includes our bodies, our selves, and our environment, all of which we seek to interweave in a complex, life-sustaining web” (Fisher and Tronto 1990, p. 40; Tronto 2013, p. 19). As such, we characterize social care as those care practices that support and strengthen people’s living situation, both individual and social, mainly by relying on social interaction and communication. Hence, social care as we conceive it includes, among others, psychology, social work, and different forms of counseling.

Existing research towards effects of social media in social care settings have, among others, focused on how social media and their contents impact people’s mental health (Sharma et al. 2020), for example how social media impact people’s bodily self-perception (Holland and Tiggemann 2016); how new forms of problematic behavior develop on social media platforms (e.g., cyberbullying, online abuse) (Willoughby 2019); and how social media can be implemented in the education of students of different social care disciplines (Franklin et al. 2016).

Moreover, research on how social media can be used in social care practices themselves has gained some traction (Chan 2016; Rosenberg et al. 2021; Cosgrove et al. 2017; Diamond and Whalen 2019). Social media enable interaction among clients seeking a peer network (Diamond and Whalen 2019; Naslund et al. 2020; Rosenberg et al. 2021) and between clients and professionals (Buzzi and Allen 2020; Megele and Buzzi 2020; Naslund et al. 2020; Rosenberg et al. 2021). Social media is also used by professionals as a way to search for and reach those in need of services (Chan 2016; Rosenberg et al. 2021) and to monitor or trace clients or ex-clients (Chan 2016; Cooner et al. 2020; Rosenberg et al. 2021). As Cooner et al. (2020, p. 148), based on their ethnographic study involving social workers that provide family and child services, indicate, the “[…] use of Facebook as part of their repertoire of everyday tools for working with families” is in the process of normalization. Additionally, the use of social media can be a means to guarantee a certain continuity of social care practices during emergency and crisis situations. For example, Rosenberg et al. (2021), based on their interview study with Israeli youth counselors and coordinators, social workers, a clinical psychologist, and senior and management personnel, indicate that therapeutic relationships “[…] that are formed online are especially relevant to […] complicated times of social distancing due to the COVID- 19 […]” pandemic. Research into the actual use of social media in social care is, however, minimal as Chan (2016), and more recently Cooner et al. (2020), indicate. Or as Chan (2016, p. 270) states the use of social media in social care is a case of “[…] rich application but weak empirical evidence […]”.

Notwithstanding, providing services via social media technologies is an ethically sensitive endeavor which entails both benefits (e.g., greater accessibility to services, cost-efficiency) and risks (e.g., loss of privacy). Moreover, it seems that existing codes of ethics do not always provide guidance for how to deal with possible ethical issues (Pascoe 2021). Clear ethical conditions, however, can strengthen the potentialities of social media use in social care. As such, there is a need to get better insight into the ethics of this use. With this systematic review, we investigate the current ethical debate on the use of social media technologies in social care practices so to better understand the ethical arguments and grounding concepts described in the ethics literature.

## Methods

The aim of this systematic review of argument-based ethics literature is to reach an exhaustive and in-depth overview of existing ethical argumentation related to social media use in social care, so as to get grounded insight into the overall debate. To meet this aim, we used a four step methodology: (1) developing ethical-conceptual questions; (2) addressing these questions by carrying out a systematic literature search for peer-reviewed academic articles via the use of a self-constructed search string; (3) assessment and inclusion of relevant articles (4) extracting and synthesizing relevant reported data (McCullough et al. 2007; McDougall 2014; Mertz et al. 2016; Sofaer and Strech 2012; Vandemeulebroucke et al. 2018). The *Preferred Reporting Items for Systematic Reviews and Meta-Analyses (PRISMA)* inspired the search and the selection process (Liberatti et al. 2009). This review was carried out by the authors who have expertise in the development of systematic reviews of ethics literature, care ethics, and bioethics (TV) and philosophy of technology (LB and TV).

### Conceptual-ethical research questions

To gain insight into the ethical debate on social media use in social care settings, it is important to study the arguments made and their grounding concepts (Vandemeulebroucke et al. 2018). As Vandemeulebroucke et al. (2018, p. 17) indicate, these concepts “[…] can be derived from traditional and/or current practices, existing ethical theories and/or what is considered to be virtuous” As ethical concepts (e.g., autonomy, privacy) frequently have different meanings depending on the context in which they are used, they can ground different, sometimes opposing, arguments. Consequently, we formulated two conceptual-ethical questions to guide the development of this review, each with its own aim:

- What are the arguments made in the ethical debate on the use of social media in social care practices?
- What are the ethical concepts grounding these arguments?

While the aim of the first question was to give an exhaustive overview of the arguments made in the ethical debate on social media use in social care, the aim of the second was to gain a deeper understanding of these arguments’ used concepts (Vandemeulebroucke et al. 2018).

### Search strategy

Based on the conceptual-ethical questions, a search string was constructed which covered three word groups. A first group referred to social media, the technology under consideration. A second group focused on the context in which social media is used, this being social care. A third group referred to the ethics dimension of the literature. The first author (TV) developed the different search strings and discussed and finalized them with the co-author (LB). Four electronic databases were queried: CINAHL, Philosopher’s Index, Web of Science, and ProQuest Database Psychology. A first search string was made to be applicable in CINAHL (Tables 1 and 2). Afterwards, it was modified to be applied to the three other databases, for example, by adjusting the qualifiers, such as the field codes, to the respective databases.

**Table 1:** Overview used databases and related search strings (Original search done on 31.01.2022)

DATABASES	WORD GROUP 1: Technology	AND	WORD GROUP 2: Setting	AND	WORD GROUP 3: Ethics	
CINAHL Timespan: 2010–2022 Date: 31.01.2022	(AB Tech* OR TI Tech* OR MH Technology OR AB “Social Media” OR TI “Social Media” OR MH “social media” OR AB mhealth OR TI mhealth OR AB telehealth OR TI telehealth OR MH telepsychiatry OR AB “Social network*” OR TI “Social network*” OR MH “Online Social networking” OR AB Internet OR TI Internet OR MH Internet OR AB “Digital tech*” OR TI “Digital tech*” OR AB “Digital service*” OR TI “Digital service*”)		(AB Psycholog* OR TI Psycholog* OR MH Psychology OR AB Psychiatr* OR TI Psychiatr* OR MH Psychiatry OR AB Psychotherap* OR TI Psychotherap* OR MH Psychotherapists OR AB “Mental Car*” OR TI “Mental Car*” OR AB “Social work” OR TI “Social work” OR MH “Social work” OR AB “Mental health*” OR TI “Mental health*” OR MH “Mental Health” OR AB “Distance counsel*” OR TI “Distance counsel*”)		(AB Ethic* OR TI Ethic* OR MH Ethics OR AB Boundar* OR TI Boundar* OR AB Philosoph* OR TI Philosoph* OR MH Philosophy OR MH “Social Media Ethical Issues” OR MH “Psychology Ethical Issues” OR MH “Social Networking Ethical Issues” OR AB Guideline* OR TI Guideline*)	1671
Philosopher’s Index (Ovid) Timespan: 2010–2022 Date: 31.01.2022	"tech*".ab,ti. or"technology".sh. or"social media".ab,ti,sh. or"mhealth".ab,ti. or"telehealth".ab,ti. or"social network*".ab,ti. or"social network".sh. or"social networking".sh. or"internet".ab,ti,sh. or"digital tech*".ab,ti. or"digital technology".sh. or"digital service*".ab,ti		"psycholog*".ab,ti. or"psychology".sh. or"psychiatr*".ab,ti. or"psychiatry".sh. or"psychotherap*".ab,ti. or"psychotherapy".sh. or"mental car*".ab,ti. or"social work".ab,ti,sh. or"mental health".ab,ti,sh. or"distance counsel*".ab,ti		"ethic*".ab,ti. or"ethics".sh. or"boundar*".ab,ti. or"philosoph*".ab,ti. or"philosophy".sh. or"guideline*".ab,ti	328
Web of Science Core Collection Timespan: 2010–2022 Date: 31.01.2022	AB = (Tech* OR"Social Media"OR mhealth OR telehealth OR telepsychiatry OR"social network*"OR"online social networking"OR internet OR"digital tech*"OR"Digital service") OR TI = (Tech* OR"Social Media"OR mhealth OR telehealth OR telepsychiatry OR"social network*"OR"online social networking"OR internet OR"digital tech*"OR"Digital service*")		AB = (Psycholog* OR Psychiatr* OR Psychotherap* OR"Mental Car*"OR"Social work"OR"mental health*"OR"distance counsel*") OR TI = (Psycholog* OR Psychiatr* OR Psychotherap* OR"Mental Car*"OR"Social work"OR"mental health*"OR"distance counsel*")		AB = (Ethic* OR Boundar* OR Philosoph* OR Guideline*) OR TI = (Ethic* OR Boundar* OR Philosoph* OR Guideline*)	4119
ProQuest Database Psychology Timespan: 2010–2022 Date: 31.01.2022	(ab(tech*) OR ti(tech*) OR su(technology) OR ab("social media") OR ti("social media”) OR su(“social media”) OR ab(mhealth) OR ti(mhealth) OR ab(telehealth) OR ti(telehealth) OR ab("social network*") OR ti("social network*") OR su("social network") OR su("social networking") OR ab(internet) OR ti(internet) OR su(internet) OR ab("digital tech*") OR ti("digital tech*") OR su("digital technology") OR ab("digital service*") OR ti("digital service*"))		(Ab(psycholog*) OR ti(psycholog*) OR su(psychology) OR ab(psychiatr*) OR ti(psychiatr*) OR su(psychiatry) OR ab(psychotherap*) OR ti(psychotherap*) OR su(psychotherapy) OR ab(mental car*) OR ti(mental car*) OR ab("social work") OR ti("social work") OR su("social work") OR ab("mental health") OR ti("mental health") OR su("mental health") OR ab("distance counsel*") OR ti("distance counsel*"))		(Ab(ethic*) OR ti(ethic*) OR su(ethics) OR ab(boundar*) OR ti(boundar*) OR ab(philosoph*) OR ti(philosoph*) OR su(philosophy) OR ab(guideline*) OR ti(guideline*))	1118
						Total: 7236

**Table 2:** Overview used databases and related search strings (Additional search done on 04.02.2024)

DATABASES	WORD GROUP 1: Technology	AND	WORD GROUP 2: Setting	AND	WORD GROUP 3: Ethics	
CINAHL Timespan: 2022–2024 Date: 04.02.2022	(AB Tech* OR TI Tech* OR MH Technology OR AB “Social Media” OR TI “Social Media” OR MH “social media” OR AB mhealth OR TI mhealth OR AB telehealth OR TI telehealth OR MH telepsychiatry OR AB “Social network*” OR TI “Social network*” OR MH “Online Social networking” OR AB Internet OR TI Internet OR MH Internet OR AB “Digital tech*” OR TI “Digital tech*” OR AB “Digital service*” OR TI “Digital service*”)		(AB Psycholog* OR TI Psycholog* OR MH Psychology OR AB Psychiatr* OR TI Psychiatr* OR MH Psychiatry OR AB Psychotherap* OR TI Psychotherap* OR MH Psychotherapists OR AB “Mental Car*” OR TI “Mental Car*” OR AB “Social work” OR TI “Social work” OR MH “Social work” OR AB “Mental health*” OR TI “Mental health*” OR MH “Mental Health” OR AB “Distance counsel*” OR TI “Distance counsel*”)		(AB Ethic* OR TI Ethic* OR MH Ethics OR AB Boundar* OR TI Boundar* OR AB Philosoph* OR TI Philosoph* OR MH Philosophy OR MH “Social Media Ethical Issues” OR MH “Psychology Ethical Issues” OR MH “Social Networking Ethical Issues” OR AB Guideline* OR TI Guideline*)	488
Philosopher’s Index (Ovid) Timespan: 2022–2024 Date: 04.02.2022	"tech*".ab,ti. or"technology".sh. or"social media".ab,ti,sh. or"mhealth".ab,ti. or"telehealth".ab,ti. or"social network*".ab,ti. or"social network".sh. or"social networking".sh. or"internet".ab,ti,sh. or"digital tech*".ab,ti. or"digital technology".sh. or"digital service*".ab,ti		"psycholog*".ab,ti. or"psychology".sh. or"psychiatr*".ab,ti. or"psychiatry".sh. or"psychotherap*".ab,ti. or"psychotherapy".sh. or"mental car*".ab,ti. or"social work".ab,ti,sh. or"mental health".ab,ti,sh. or"distance counsel*".ab,ti		"ethic*".ab,ti. or"ethics".sh. or"boundar*".ab,ti. or"philosoph*".ab,ti. or"philosophy".sh. or"guideline*".ab,ti	39
Web of Science Core Collection Timespan: 2022–2024 Date: 04.02.2022	AB = (Tech* OR"Social Media"OR mhealth OR telehealth OR telepsychiatry OR"social network*"OR"online social networking"OR internet OR"digital tech*"OR"Digital service") OR TI = (Tech* OR"Social Media"OR mhealth OR telehealth OR telepsychiatry OR"social network*"OR"online social networking"OR internet OR"digital tech*"OR"Digital service*")		AB = (Psycholog* OR Psychiatr* OR Psychotherap* OR"Mental Car*"OR"Social work"OR"mental health*"OR"distance counsel*") OR TI = (Psycholog* OR Psychiatr* OR Psychotherap* OR"Mental Car*"OR"Social work"OR"mental health*"OR"distance counsel*")		AB = (Ethic* OR Boundar* OR Philosoph* OR Guideline*) OR TI = (Ethic* OR Boundar* OR Philosoph* OR Guideline*)	1822
ProQuest Database Psychology Timespan: 2022–2024 Date: 04.02.2022	(ab(tech*) OR ti(tech*) OR su(technology) OR ab("social media") OR ti("social media”) OR su(“social media”) OR ab(mhealth) OR ti(mhealth) OR ab(telehealth) OR ti(telehealth) OR ab("social network*") OR ti("social network*") OR su("social network") OR su("social networking") OR ab(internet) OR ti(internet) OR su(internet) OR ab("digital tech*") OR ti("digital tech*") OR su("digital technology") OR ab("digital service*") OR ti("digital service*"))		(Ab(psycholog*) OR ti(psycholog*) OR su(psychology) OR ab(psychiatr*) OR ti(psychiatr*) OR su(psychiatry) OR ab(psychotherap*) OR ti(psychotherap*) OR su(psychotherapy) OR ab(mental car*) OR ti(mental car*) OR ab("social work") OR ti("social work") OR su("social work") OR ab("mental health") OR ti("mental health") OR su("mental health") OR ab("distance counsel*") OR ti("distance counsel*"))		(Ab(ethic*) OR ti(ethic*) OR su(ethics) OR ab(boundar*) OR ti(boundar*) OR ab(philosoph*) OR ti(philosoph*) OR su(philosophy) OR ab(guideline*) OR ti(guideline*))	488
						Total: 2837

The search was carried out on January 31, 2022 (Table 1). As the original review process took around two years, an additional search was done on February 4, 2024 (Table 2), to ensure that newly published relevant articles were not missed. Although social networking sites have existed from the 1990’s onwards, social media as a concept as well as its use only gradually became widespread among the public with the rise of online platforms such as Facebook (2004), YouTube (2005), Twitter (now X) (2006) (Van Dyck 2013), and more recently TikTok (2016) (Montag et al. 2021). Hence, reasoning that it would take some time for these social media to be implemented in social care practice, the search was limited to publications from 2010 onwards. Hence, the first search was limited to the period 2010–2022, the second to the period 2022–2024.

### Inclusion and exclusion criteria

Search results were compiled in a reference manager (Endnote™ version 20.2.1.15749. Clarivate Analytics, Philadelphia, PA, USA). Before applying the specific inclusion and exclusion criteria, results were screened for their publication type. Results that were not indicated as being peer-reviewed articles were removed. After, duplicates were removed.

Inclusion and exclusion criteria, related to the results’ “Publication Type”, “Topic”, “Research Method” and “Language” were then systematically and sequentially applied to titles, abstracts, and full texts independently by the two authors (Liberatti et al. 2009). As indicated, to be considered eligible, publications had to be peer-reviewed articles (Publication Type). Additionally, articles had to focus on the ethics of the use of social media in social care (Topic) and had to consist of ethical argumentation in relation to the use of social media in social care (Research Method). Finally, articles had to be written in English, Dutch, German, or French (Table 3).

**Table 3:** Inclusion and exclusion criteria for selecting publications for data extraction and synthesis

Inclusion criteria	Exclusion criteria
Publication type	
International scientific peer-reviewed articles	All other than international scientific peer-reviewed articles, for example: Reviews Editorials Grey literature Commentaries Opinion papers Books Book chapters Theses
Topic	
Social media technology	All other than social media technology, for example: Email; SMS-messaging: Care apps
Social care settings: Psychology, Psychiatry, Social Work, Rehabilitation Counselling	All others than social-care settings, for example: Healthcare settings; Research settings; Educational settings; Labor/Work settings; Leisure settings
Ethics literature	All other than ethics literature
Research method	
Ethical argumentation	All other than ethical argumentation, for example: Qualitative empirical research; Quantitative empirical research; Mere mentioning of ethical issues
Language	
English	All other than included languages
Dutch	
French	
German	

**Fig. 1:**
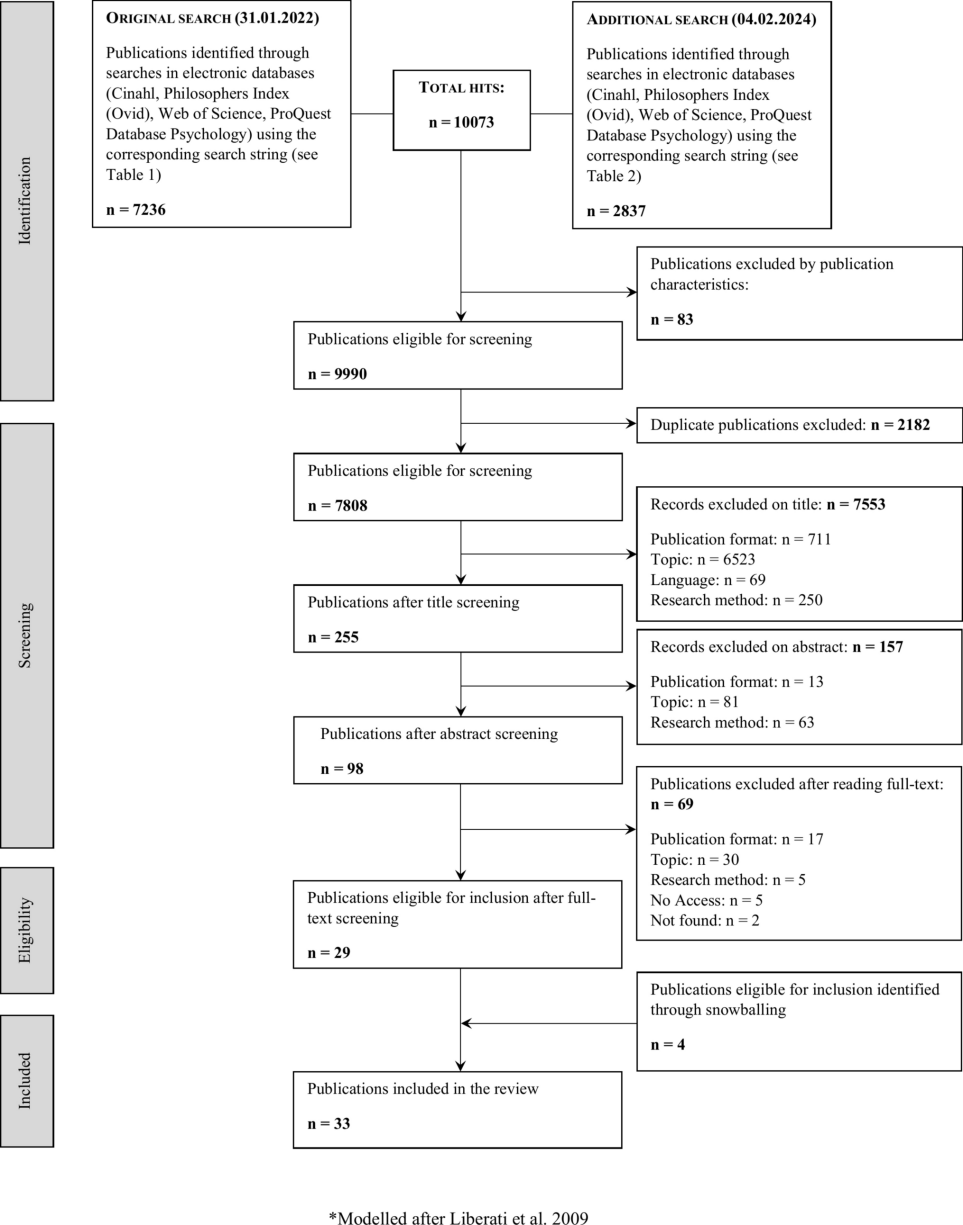
Flowchart overview screening process (Modelled after Liberati et al. 2009)

During each sequence of title, abstract, and full text screening, the results of the two authors were compared. Those publications of which eligibility was uncertain were discussed until consensus was reached. Finally, by checking the reference lists of those articles that were deemed eligible to be included in this review, so-called snowballing, the authors identified those articles that were not identified by the conducted search. Figure 1 presents the search and screening processes.

### Data extraction and synthesis

Extraction of relevant data was executed by use of a custom-made extraction tool which was inspired by the tool developed by Vandemeulebroucke et al. (2022c) but further amended after several readings of the included literature (Additional file 1).

#### Data extraction tool

The tool consists of part focusing on publication characteristics and part focusing on the publication content. The part focusing on publication characteristics covered: (1) Language in which the article is written; (2) The country of the first author’s affiliation; (3) The first author’s profession or research discipline; (4) Research field which the article places itself in; (5) Research focus of the publishing journal; (6) Year of publication.

The part of the tool focusing on the content was directed at the articles’ ethical arguments and concepts. After an iterative process of multiple readings of the included articles, 11 themes to guide the analysis of the reported ethical arguments and used ethical concepts were distilled: (1) Benefits of social media; (2) Risks of social media; (3) Relations, limits and boundaries; (4) Privacy, confidentiality, and trust; (5) Documentation and records; (6) Competency; (7) Problematic behavior and client suitability; (8) Consultation and referral; (9) Informed consent; (10) Identity and image; (11) Others. These 11 themes were joined with two guiding questions on how social media as a technology was perceived and which ethical approach or theory grounded the articles. The final version of the data extraction tool was used to analyze the included literature.

#### Extraction and synthesis of data

The analysis and synthesis of data was inspired by the five preparatory steps of the coding process of the Qualitative Analysis Guide of Leuven (QUAGOL) (Dierckx de Casterlé et al. 2012). First, both authors thoroughly familiarized themselves with the content of the included articles by several readings of the full text. This familiarization enabled the authors to further develop the data extraction tool by including the 11 themes of focus which in a second step was used to obtain all relevant data reported in the included articles. Of each of these articles, a conceptual scheme was made (Additional file 2). Third, by comparing the conceptual schemes, common publication characteristics and content characteristics were identified on a per-characteristic basis. Fourth, tables were drafted by the first author (TV) summarizing the similarities and differences between the conceptual schemes. These tables were discussed with the co-author (LB) and finetuned. Fifth, these tables guided the reporting of the results in this article (Additional file 3).

## Results

Thirty-three articles were finally included in this systematic review (Table 4). Table 5 gives a structured overview of their publication characteristics. Here, we focus on the articles’ content (Table 4). The current ethics of social media use in social care mainly focusses on the particular therapeutic relationship between professionals and clients, or on one or both parties in it. Moreover, the analysis made clear that, in the included articles, ‘social media’ is used in three manners: 1) Social media was discussed in general terms, with most of these articles mentioning some particular social media by way of example (e.g., Facebook, Instagram, LinkedIn, Youtube) (Baier 2019; Barnett 2019; Belkofer and McNutt 2011; Boddy and Dominelli 2017; Bratt 2010; Crtalic et al. 2015; Frankish et al. 2012; Froehlich et al. 2023; Hartley et al. 2015; Jordan et al. 2014; Kaluzeviciute 2020; Kaplan et al. 2011; Kellen et al. 2015; Kolmes 2012; Lannin and Scott 2013; Smith et al. 2023; 2) social media was discussed as part of or as an example of a greater analysis of technology use in social care (e.g., general technology use, telepsychology, teletherapy, mobile technology, internet use, telerehabilitation, digital technology), with most articles also mentioning particular social media as examples (Barsky 2017; Cooper et al. 2019; Dombo et al. 2014; Drum and Littleton 2014; Edwars-Stewart et al. 2019; Fantus and Mishna 2013; Gabbard et al. 2011; Holmes and Reid 2018; Kaslow et al. 2011; Mattison 2018; Nicholson 2011; Reamer 2013, 2015, 2017, 2018; Steiner 2021; or 3) social media was discussed with a focus on one particular social media (e.g., Facebook) (Lehavot et al. 2012).

**Table 4:** Overview of included studies (n = 33)

	Article	Location	Focus of the article	Social media of interest	Ethical principles/values and ethics sources^a,b^	Benefits and ethical vulnerabilities with social media use^c^
1	Baier (2019)	United States of America (USA)	Psychology	Social media discussed in general terms Mentioned examples of social media: Facebook, Twitter, Instagram	Ethical principles/values: ∙ Beneficence ∙ Non-maleficence ∙ Fidelity & responsibility ∙ Integrity ∙ Justice ∙ Respect for people’s rights and dignities Ethics sources: ∙ Ethical Principles of Psychologists and Code of Conduct (American Psychological Association (APA) 2002)	Benefits: ∙ None mentioned Ethical vulnerabilities ∙ Relationships, boundaries, and limits ∙ Searches ∙ Privacy, confidentiality, and trust ∙ Identity and image
2	Barnett (2019)	USA	Psychology	Social media discussed in general terms Mentioned examples of social media: Facebook	Ethical principles/values: ∙ Not specified Ethics sources: ∙ Code of Ethics of the American Counseling Association (ACA 2014) ∙ Ethical Principles of Psychologist and Code of Conduct (APA 2017)	Benefits: ∙ None mentioned Ethical vulnerabilities: ∙ Relations, boundaries, and limits ∙ Privacy, confidentiality, and trust ∙ Competency and client suitability ∙ Informed consent
3	Barsky (2017)	USA	Social work	Social media as part of greater analysis of technology use Mentioned examples of social media: Facebook, Youtube, Twitter	Ethical principles/values: ∙ Respect for dignity and worth of all people ∙ Integrity ∙ Social justice Ethics sources: ∙ Ethics Code of the National Association Social Workers (NASW 2008) (USA) ∙ Draft Standards for Technology and Social Work Practice. Developed in Conjunction with Council on Social Work Education, Association of Social Work Boards, and Clinical Social Work Association (NASW 2016)^d^	Benefits: ∙ Increased accessibility to services Ethical vulnerabilities: ∙ Relations, boundaries, and limits ∙ Searches ∙ Privacy, confidentiality, and trust ∙ Documentation and records ∙ Competency and client suitability ∙ Consultation and referral ∙ Identity and image ∙ Informed consent
4	Belkofer and McNutt (2011)	USA	Art therapy	Social media discussed in general terms Mentioned examples of social media: Facebook	Ethical principles/values: ∙ Not specified Ethics sources: ∙ Not specified	Benefits: ∙ None mentioned Ethical vulnerabilities: ∙ Relations, boundaries, and limits ∙ Searches ∙ Privacy, confidentiality, and trust ∙ Competency and client suitability ∙ Identity and image ∙ Informed consent
5	Boddy and Dominelli (2017)	Australia	Social work	Social media discussed in general terms Mentioned examples of social media: Facebook	Ethical principles/values: ∙ Human rights ∙ Social justice ∙ Integrity ∙ Competence ∙ Respect ∙ Dignity ∙ Social critique Ethics sources: ∙ Not specified	Benefits: ∙ Increased therapeutic value due to clients’ increased feeling of empowerment Ethical vulnerabilities: ∙ Relations, boundaries, and limits ∙ Privacy, confidentiality, and trust ∙ Competency and client suitability ∙ Consultation and referral ∙ Identity and image ∙ Informed consent
6	Bratt (2010)	Canada	Psychology	Social media discussed in general terms Mentioned examples of social media: Facebook, Myspace, Twitter	Ethical principles/values: ∙ Integrity ∙ Autonomy ∙ (Client) Self-determination ∙ Right to live one’s own life ∙ Confidentiality Ethics sources: ∙ Not specified	Benefits: ∙ None mentioned Ethical vulnerabilities: ∙ Relations, boundaries, and limits ∙ Searches ∙ Privacy, confidentiality, and trust ∙ Documentation and records ∙ Competency and client suitability ∙ Identity and image ∙ Informed consent
7	Cooper and Barnwell (2019)	USA	Psychology	Social media as part of greater analysis of technology use Mentioned examples of social media: Facebook, Instagram, Snapchat	Ethical principles/values: ∙ Social justice ∙ Equity ∙ Informed Consent/autonomy ∙ Confidentiality Ethics sources: ∙ Not specified	Benefits: ∙ Increased accessibility to services ∙ Increased feelings of emotional safety and comfort ∙ Continuous availability of support ∙ Increased therapeutic value due to clients’ increased feeling of empowerment Ethical vulnerabilities: ∙ Relations, boundaries, and limits ∙ Searches ∙ Privacy, Confidentiality, and trust ∙ Competency and client suitability ∙ Consultation and referral ∙ Identity and image ∙ Informed consent
8	Crtalic et al. (2015)	USA	Rehabilitation counseling	Social media discussed in general terms Mentioned examples of social media: Facebook, Linkedin, Google Community, Instagram	Ethical principles/values: ∙ Boundaries ∙ Confidentiality and Social Media Ethics sources: ∙ ACA Code of Ethics (ACA 2014) ∙ Code of Professional Ethics for Rehabilitation Counselors (Commission on Rehabilitation Counselor Certification (CRCC) 2009) ∙ Social Media Policy for Certificants and the general Public (CRCC 2012)^d^	Benefits: ∙ Increased accessibility to services ∙ Increased feelings of emotional safety and comfort ∙ Reduce clients’ levels of anxiety, shyness, and fear Ethical vulnerabilities: ∙ Relations, boundaries, and limits ∙ Privacy, confidentiality, and trust ∙ Competency and client suitability ∙ Consultation and referral ∙ Identity and image ∙ Informed consent
9	Dombo et al. (2014)	USA	Social work	Social media as part of greater analysis of technology use Mentioned examples of social media: Facebook, LinkedIn, Twitter, Instagram	Ethical principles/values ∙ Integrity v Responsibility ∙ Autonomy/informed consent/privacy Ethics sources: ∙ Code of Ethics (National Association of Social Workers (NASW) 2008) ∙ NASW & ASWB Standards for Technology and Social Work Practice (National Association of Social Workers & Association of Social Work Boards (NASW and ASWB) 2005)^d^	Benefits: ∙ Increased accessibility to services ∙ Increased feelings of emotional safety and comfort Ethical vulnerabilities: ∙ Relations, boundaries, and limits ∙ Searches ∙ Privacy, confidentiality, and trust ∙ Competency and client suitability ∙ Consultation and referral ∙ Identity and image ∙ Informed consent
10	Drum and Littleton (2014)	USA	Psychology	Social media as part of greater analysis of technology use Mentioned examples of social media: Facebook, Twitter	Ethical principles/values ∙ Not specified Ethics sources: ∙ Not specified	Benefits: ∙ None mentioned Ethical vulnerabilities: ∙ Relations, boundaries, and limits ∙ Privacy, Confidentiality, and trust ∙ Documentation and records ∙ Competency and client suitability ∙ Identity and image ∙ Informed consent
11	Edwards-Stewart et al. (2019)	USA	Psychology	Social media as part of greater analysis of technology use Mentioned examples of social media: None	Ethical principles/values ∙ Beneficence ∙ Non-maleficence ∙ Justice Ethics sources: ∙ Ethical Principles of Psychologists and Code of Conduct (APA 2017)	Benefits: ∙ Increased accessibility to services Ethical vulnerabilities: ∙ Privacy, confidentiality, and trust ∙ Competency and client suitability ∙ Informed consent
12	Fantus and Mishna (2013)	Canada	Social work	Social media as part of greater analysis of technology use Mentioned examples of social media: Facebook, LinkedIn, Myspace	Ethical principles/values ∙ Confidentiality ∙ Boundaries ∙ Competence Ethics sources: ∙ Code of Ethics and Standards of Practice Handbook (Ontaria College of Social Workers and Social Service Workers (ACSWSSW 2008) ∙ Code of Ethics (NASW 2013) ∙ Code of Ethics (Canadian Association of Social Workers (CASW) 2005)	Benefits: ∙ Increased accessibility to services Ethical vulnerabilities: ∙ Relations, boundaries, and limits ∙ Privacy, confidentiality, and trust ∙ Competency and client suitability ∙ Informed consent
13	Frankish et al. (2012)	Australia	Psychiatry	Social media discussed in general terms Mentioned examples of social media: Facebook, Myspace, Linkedin, Twitter, Youtube	Ethical principles/values: ∙ Confidentiality ∙ Privacy ∙ Therapeutic relationship ∙ Professionalism ∙ Online image Ethics sources: ∙ Not specified	Benefits: ∙ Increased flexible and spontaneous interaction between professionals and clients Ethical vulnerabilities: ∙ Relations, boundaries, and limits ∙ Searches ∙ Privacy, confidentiality, and trust ∙ Documentation and records ∙ Competency and client suitability ∙ Consultation and referral ∙ Identity and image ∙ Informed consent
14	Froehlich et al. (2023)	USA	Rehabilitation counseling	Social media discussed in general terms Mentioned examples of social media: Youtube, Facebook, Instagram, Twitter, LinkedIn	Ethical principles/values: ∙ Not specified Ethics sources: ∙ Code of Ethics for Professional Rehabilitation Counselors (CRCC 2017) ∙ Social Media Policy (CRCC 2021) ∙ Code of Ethics for Professional Rehabilitation Counselors (CRCC 2023)	Benefits: ∙ Increased feelings of emotional safety and comfort ∙ Increased therapeutic value due to clients’ increased feeling of empowerment Ethical vulnerabilities: ∙ Relations, boundaries, and limits ∙ Privacy, confidentiality, and trust ∙ Competency and client suitability ∙ Consultation and referral ∙ Identity and image ∙ Informed consent
15	Gabbard et al. (2011)	USA	Psychiatry	Social media as part of greater analysis of technology use Mentioned examples of social media: Facebook, Myspace	Ethical principles/values: ∙ Respect for autonomy ∙ Beneficence ∙ Nonmaleficence ∙ Justice Ethics sources: ∙ Principlism (Beauchamp 2009)	Benefits: ∙ None mentioned Tension: ∙ Relations, boundaries, and limits ∙ Searches ∙ Privacy, confidentiality, and trust ∙ Competency and client suitability ∙ Identity and image
16	Hartley et al. (2015)	USA	Rehabilitation counseling	Social media discussed in general terms Mentioned examples of social media: Twitter, Facebook, LinkedIn, Youtube, blogs, wikis	Ethical principles/values: ∙ Informed consent ∙ Privacy ∙ Competence ∙ Accessibility ∙ Boundaries Ethics sources: ∙ CRC/CCRC Code of Ethics (CRCC 2010)^d^	Benefits: ∙ Increased accessibility to services ∙ Increased therapeutic value due to clients’ increased feeling of empowerment ∙ Avoidance of public stigma Ethical vulnerabilities: ∙ Relations, boundaries, and limits ∙ Privacy, confidentiality, and trust ∙ Competency and client suitability ∙ Informed consent
17	Holmes and Reid (2018)	USA	Rehabilitation counseling	Social media as part of greater analysis of technology use Mentioned examples of social media: Facebook	Ethical principles/values: ∙ Not specified Ethics sources: ∙ ACA Code of Ethics (ACA 2014) ∙ Technology, social media and distance counseling considerations (CRCC 2016)^d^ ∙ Code of Professional Ethics for Rehabilitation Counselors (CRCC 2017) ∙ Policy regarding The Provision of Distance Professional Services (National Board of Certified Counselors (NBCC) 2016)^d^	Benefits: ∙ Increased feelings of emotional safety and comfort Ethical vulnerabilities: ∙ Relation, boundaries, and limits ∙ Searches ∙ Privacy, confidentiality, and trust ∙ Documentation and records ∙ Competency and client suitability ∙ Identity and image ∙ Informed consent
18	Jordan et al. (2014)	USA	Psychology	Social media discussed in general terms Mentioned examples of social media: Facebook, Myspace, Twitter, Youtube	Ethical principles/values: ∙ Not specified Ethics sources: ∙ AAMFT Code of Ethics (American Association for Marriage and Family Therapy (AAMFT) 2011)^d^ ∙ ACA Code of Ethics (ACA 2005) ∙ Ethical Principles of Psychologists and Code of Conduct (APA 2010)^d^ ∙ NASW Code of Ethics (NASW 2008)	Benefits: ∙ Increased accessibility to services Ethical vulnerabilities: ∙ Relations, boundaries, and limits ∙ Searches ∙ Privacy, confidentiality, and trust ∙ Competency and client suitability ∙ Consultation and referral ∙ Informed consent
19	Kaluzeviciute (2020)	United Kingdom (UK)	Psychology	Social media discussed in general terms Mentioned examples of social media: Facebook, Twitter, Instagram, LinkedIn	Ethical principles/values: ∙ Therapeutic neutrality ∙ Therapeutic anonymity ∙ Therapeutic relationship Ethics sources: ∙ Not specified	Benefits: ∙ None mentioned Ethical vulnerabilities: ∙ Relations, boundaries, and limits ∙ Searches ∙ Privacy, confidentiality, and trust ∙ Identity and image
20	Kaplan et al. (2011)	USA	Psychology	Social media discussed in general terms Mentioned examples of social media: Facebook, Twitter, LinkedIn	Ethical principles/values: ∙ Confidentiality ∙ Confirmability ∙ Boundaries ∙ Informed consent ∙ Patient’s best interest Ethics sources: ∙ Code of Ethics (ACA 2005)	Benefits: ∙ None mentioned Ethical vulnerabilities: ∙ Relations, boundaries, and limits ∙ Searches ∙ Privacy, confidentiality, and trust ∙ Identity and image ∙ Informed consent
21	Kaslow et al. (2011)	USA	Psychology	Social media as part of greater analysis of technology use Mentioned examples of social media: Facebook, Myspace, Twitter	Ethical principles/values: ∙ Respect for autonomy ∙ Nonmaleficence ∙ Beneficence ∙ Justice ∙ Confidentiality ∙ Informed consent ∙ Privacy ∙ Trust in relationships ∙ Best interest of clients Ethics sources: ∙ Principlism (Beauchamp and Childress 2009) ∙ Ethical Principles of Psychologists and Code of Conduct (APA 2002)	Benefits: ∙ None mentioned Ethical vulnerabilities: ∙ Relations, boundaries, and limits ∙ Searches ∙ Privacy, confidentiality, and trust ∙ Competency and client suitability ∙ Consultation and referral ∙ Identity and image ∙ Informed consent
22	Kellen et al. (2015)	USA	Psychology	Social media discussed in general terms Mentioned examples of social media: Facebook, Twitter, LinkedIn, Instagram, Pinterest, Kik	Ethical principles/values: ∙ Responsibility to clients ∙ Confidentiality Ethics sources: ∙ AAMFT Code of Ethics (AAMFT 2012)^d^	Benefits: ∙ Increased accessibility to services ∙ Increased therapeutic value due to clients’ increased feeling of empowerment ∙ Avoidance of public stigma Ethical vulnerabilities: ∙ Relations, boundaries, and limits ∙ Privacy, confidentiality, and trust ∙ Competency and client suitability ∙ Consultation and referral ∙ Identity and image ∙ Informed consent
23	Kolmes (2012)	USA	Psychology	Social media discussed in general terms Mentioned examples of social media: Facebook, Twitter, LinkedIn, Myspace, Youtube	Ethical principles/values: ∙ Not specified Ethics sources: ∙ Ethical Principles of Psychologists and Code of Conduct (APA 2010)^d^	Benefits: ∙ None mentioned Ethical vulnerabilities: ∙ Relations, boundaries, and limits ∙ Searches ∙ Privacy, confidentiality, and trust ∙ Competency and client suitability ∙ Identity and image ∙ Informed consent
24	Lannin and Scott (2013)	USA	Psychology	Social media discussed in general terms Mentioned examples of social media: Facebook, Myspcace, LinkedIn, Wordpress, Blogger, Wikipedia, Youtube, Flickr, Digg, Last.fm, Yahoo Messenger, Google Talk, Skype, Twitter, Tumbler, Posterous, Friendfeed, Lifestream, Liverstream, Second Life, There	Ethical principles/values: ∙ Beneficence ∙ Nonmaleficence ∙ Integrity Ethics sources: ∙ Principlism (Beauchamp and Childress 2001) ∙ ACA Code of Ethics (ACA 2005) ∙ American Medical Association Policy: Professionalism in the Use of Social Media (American Medical Association (AMA), 2010)^d^ ∙ Ethical Principles of Psychologists and Code of Conduct (APA 2010)^d^ ∙ Ethical Guidelines for Psychologists providing Psychological Services via Electronic Media (Canadian Psychological Association (CPA) 2008)^d^	Benefits: ∙ None mentioned Ethical vulnerabilities: ∙ Relations, boundaries, and limits ∙ Searches ∙ Privacy, confidentiality, and trust ∙ Competency and client suitability ∙ Informed consent
25	Lehavot et al. (2012)	USA	Psychology	A particular social media was discussed: Facebook	Ethical principles/values: ∙ Beneficence ∙ Nonmaleficence ∙ Privacy ∙ Confidentiality ∙ Informed consent ∙ Judgement ∙ Trust ∙ Therapeutic relationship Ethics sources: ∙ Principlism (Beauchamp and Childress 2009) ∙ Ethical Principles of Psychologists and Code of Conduct (APA 2002)	Benefits: ∙ Continuous availability of support Ethical vulnerabilities: ∙ Relations, boundaries, and limits ∙ Searches ∙ Privacy, confidentiality, and trust ∙ Identity and image ∙ Informed consent
26	Mattison (2018)	USA	Social work	Social media as part of greater analysis of technology use Mentioned examples of social media: Facebook, Twitter, Instagram	Ethical principles/values: ∙ Not specified Ethics sources: ∙ None specified	Benefits: ∙ Increased feelings of emotional safety and comfort ∙ Increased therapeutic value due to clients’ increased feeling of empowerment ∙ Increased feelings of privacy Ethical vulnerabilities: ∙ Relations, boundaries, and limits ∙ Searches ∙ Privacy, confidentiality, and trust ∙ Documentation and records ∙ Competency and client suitability ∙ Identity and image ∙ Informed consent
27	Nicholson (2011)	Canada	Psychology	Social media as part of greater analysis of technology use Mentioned examples of social media: Facebook, Myspace, LinkedIn	Ethical principles/values: ∙ Dignity of Persons ∙ Integrity in Relationships Ethics sources: ∙ Canadian Code of Ethics for Psychologists (3rd ed.) (Canadian Psychological Association (CPA) 2000)	Benefits: ∙ None mentioned Ethical vulnerabilities: ∙ Relations, boundaries, and limits ∙ Searches ∙ Privacy, confidentiality, and trust ∙ Documentation and records ∙ Competency and client suitability ∙ Informed consent
28	Reamer (2013)	USA	Social work	Social media as part of greater analysis of technology use Mentioned examples of social media: Facebook, LinkedIn	Ethical principles/values: ∙ Competence ∙ Informed consent ∙ Privacy and confidentiality ∙ Boundaries ∙ Dual relationships ∙ Conflict of interest Ethics sources: ∙ Codes of Ethics of the National Association of Social Workers (NASW 2008) ∙ NASW and ASWB Standards for Technology and Social Work Practice (NASW and ASWB 2005)^d^	Benefits: ∙ Increased accessibility to services ∙ Reduce clients’ levels of anxiety, shyness, and fear ∙ Continuous availability of support Ethical vulnerabilities: ∙ Relations, boundaries, and limits ∙ Privacy, confidentiality, and trust ∙ Documentation and records ∙ Competency and client suitability ∙ Consultation and referral ∙ Identity and image ∙ Informed consent
29	Reamer (2015)	USA	Social work	Social media as part of greater analysis of technology use Mentioned examples of social media: Facebook, LinkedIn	Ethical principles/values: ∙ Informed consent ∙ Privacy and confidentiality ∙ Boundaries ∙ Dual relationships ∙ Conflicts of interest ∙ Competence ∙ Collegial relationships Ethics sources: ∙ Code of Ethics of the National Association of Social Workers (NASW 2008)	Benefits: ∙ Reduce clients’ levels of anxiety, shyness, and fear ∙ Continuous availability of support ∙ Increased therapeutic value due to clients’ increased feeling of empowerment ∙ Increased feelings of privacy Ethical vulnerabilities: ∙ Relations, boundaries, and limits ∙ Searches ∙ Privacy, confidentiality, and trust ∙ Documentation and records ∙ Competency and client suitability ∙ Identity and image ∙ Informed consent
30	Reamer (2017)	USA	Social work	Social media as part of greater analysis of technology use Mentioned examples of social media: Facebook, LinkedIn	Ethical principles/values: ∙ Competence ∙ Informed consent ∙ Privacy and confidentiality ∙ Boundaries ∙ Dual relationships ∙ Conflict of interest ∙ Collegial relationships Ethics sources: ∙ Model regulatory standards for technology and social work practice (ASWB International Technology Task Force 2015)	Benefits: ∙ Increased accessibility to services ∙ Continuous availability of support ∙ Increased therapeutic value due to clients’ increased feeling of empowerment ∙ Increased feelings of privacy Ethical vulnerabilities: ∙ Relations, boundaries, and limits ∙ Searches ∙ Privacy, confidentiality, and trust ∙ Documentation and records ∙ Competency and client suitability ∙ Consultation and referral ∙ Identity and image ∙ Informed consent
31	Reamer (2018)	USA	Social work	Social media as part of greater analysis of technology use Mentioned examples of social media: None	Ethical principles/values: ∙ Competence ∙ Informed consent ∙ Privacy & confidentiality ∙ Boundaries ∙ Dual relationships ∙ Conflicts of interest ∙ Collegial relationships Ethics sources: ∙ Code of ethics (AAMFT 2015) ∙ Code of ethics (ACA 2014) ∙ Guidelines for the Practice of Telepsychology (APA 2013) ∙ NASW, ASWB, CSWE and CSWA Standards for Technology in Social Work Practice. (National Association of Social Workers, Association of Social Work Boards (ASWB), Council on Social Work Education (CSWE), and Clinical Social Work Association (CSWA) 2017)	Benefits: ∙ Increased accessibility to services ∙ Continuous availability of support Ethical vulnerabilities: ∙ Relations, boundaries, and limits ∙ Search ∙ Privacy, confidentiality, and trust ∙ Documentation and records ∙ Competency and client suitability ∙ Consultation and referral ∙ Identity and image
32	Smith and Hunter (2023)	USA	Psychology	Social media discussed in general terms Mentioned examples of social media: Facebook, Snapchat, twitter, Instagram, TikTok	Ethical principles/values: ∙ Competence ∙ Relationships ∙ Avoiding harm ∙ Informed consent ∙ Privacy and confidentiality ∙ Psychologist presence Ethics sources: ∙ Ethical Principles of Psychologists and Code of Conduct (2002, Amended Effective June 1, 2010, and January 1, 2017) (APA 2017)	Benefits: ∙ None mentioned Ethical vulnerabilities: ∙ Relations, boundaries, and limits ∙ Privacy, confidentiality, and trust ∙ Competency and client suitability ∙ Identity and image ∙ Informed consent
33	Steiner (2021)	Switzerland	Social work	Social media as part of greater analysis of technology use Mentioned examples of social media: None	Ethical principles/values: ∙ Responsibility ∙ Social work ethos ∙ Social justice ∙ Inclusion ∙ Relationship Ethics sources: ∙ Actor-network-theory (ANT) (Latour 2007) ∙ Deliberative ethics (Habermas 1992)	Ethics of responsibility ∙ Social work to actively shaping the digital transformation according to social work values

^a^In some articles, clear distinctions between ethical principles/values and perceived ethical benefits and vulnerabilities could not be made

^b^Of some of the ethics sources used by the included articles, the corresponding reference could not be found anymore. If the reference could not be found, we indicated this by superscript ‘d’; the full reference is nonetheless included in the reference list

^c^This column is based on the conceptual analysis of the authors

A more fine-grained and detailed analysis of the different benefits and ethical vulnerabilities addressed by the individual articles can be found in Supplementary File 3

^d^Reference not found

**Table 5:** Report characteristics of included literature

Included literature n = 33	
Language	
English	n = 33
Country of 1 st author	
United States of America	n = 26
Canada	n = 3
Australia	n = 2
United Kingdom	n = 1
Switzerland	n = 1
Professional background1^st^ author	
Psychology	n = 13
Social Work	n = 10
Rehabilitation	n = 4
Psychiatry	n = 3
Family, Couple, Marriage Therapy	n = 2
Art Therapy	n = 1
Focus article	
Psychology	n = 13
Social Work	n = 10
Rehabilitation	n = 4
Psychiatry	n = 3
Family, Couple, Marriage Therapy	n = 2
Art Therapy	n = 1
Focus of Journal	
Psychology	n = 12
Social Work	n = 8
Rehabilitation	n = 4
Psychiatry	n = 3
Family, Couple, Marriage Therapy	n = 2
Ethics	n = 2
Technology	n = 1
Art Therapy	n = 1
Year of Publication	
2020–2024	n = 4
2015–2019	n = 14
2010–2014	n = 15

Of the 11 themes that were included in the data extraction tool, some had to be adjusted while new ones had to be developed. In the end, the analysis identified nine themes related to the ethics of social media use that structure this result section: (1) Benefits of social media; (2) Relations, limits, and boundaries; (3) Searches (4) Privacy, confidentiality, and trust; (5) Documentation and records; (6) Competency and client suitability; (7) Consultation and referral; (8) Informed consent; and (9) Identity and image.

### Benefits of social media

It became clear that in much of the included literature, before the specific ethical vulnerabilities of social media use are discussed, attention is paid to their possible benefits. Many authors refer to the possibility of increased accessibility of services. This could especially be the case for clients that are disabled, sick or live remotely (Barsky 2017; Cooper et al. 2019; Crtalic et al. 2015; Dombo et al. 2014; Fantus and Mishna 2013; Hartley et al. 2015; Reamer 2013, 2017, 2018). Social media use could increase feelings of emotional safety and comfort (Cooper et al. 2019; Crtalic et al. 2015; Dombo et al. 2014; Froehlich et al. 2023; Holmes and Reid 2018; Mattison 2018), as clients would not need to have to go to the professional’s practice office. Moreover, it would make interaction between professionals and clients more flexible and spontaneous (Frankish et al. 2012) which could reduce clients’ levels of anxiety, shyness, and fear (Barsky 2017; Crtalic et al. 2015; Reamer 2013, 2015). The fact that social media can be used continuously can lead to feelings of comfort as help and support could be provided to the client after hours, for example during a crisis (Cooper et al. 2019; Lehavot et al. 2012; Reamer 2013, 2015, 2017, 2018). Finally, the use of social media could make social care practices more cost-effective and so more financially accessible (Cooper et al. 2019; Crtalic et al. 2015; Edwards-Stewart et al. 2019; Jordan et al. 2014; Kellen et al. 2015; Reamer 2018). Some authors question this possible increase in financial accessibility as technologies necessary to use social media can induce higher costs and so create unequal access (Hartley et al. 2015; Holmes and Reid 2018; Mattison 2018; Reamer 2018).

Nevertheless, the potentially greater accessibility of services can lead to an increased feeling of empowerment among clients and consequently, an increased therapeutic value of given services (Boddy and Dominelli 2017; Cooper et al. 2019; Froehlich et al. 2023; Hartley et al. 2015; Kellen et al. 2015; Mattison 2018; Reamer 2015, 2017). Social media enable clients to interact with professionals outside an office context by which clients can experience increased feelings of privacy because, for example, clients do not need to wait in a waiting room risking being recognized (Mattison 2018; Reamer 2015, 2017). Moreover, clients could avoid the public stigma (Hartley et al. 2015; Kellen et al. 2015) which is still attached to some forms of social care such as mental healthcare.

### Relations, boundaries, and limits

Almost all articles focus on how social media will influence the therapeutic relationship between clients and professionals, especially on how boundaries securing the relationship will be redrawn. Boundaries are those rules and agreements which help to differentiate therapeutic from other relationships (e.g., business, social) and which enable it to flourish (Drum and Littleton 2014; Frankish et al. 2012). Basic boundaries are for example that therapeutic relationships are limited in time, context dependent, and contain certain levels of anonymity (Crtalic et al. 2015; Drum and Littleton 2014; Kaluzeviciute 2020).

Many authors point to the increased risk of boundary blurring, confusion, and crossing between professionals, clients, and/or former clients by social media use (Barsky 2017; Boddy and Dominelli 2017; Bratt 2010; Crtalic et al. 2015; Drum and Littleton 2014; Frankish et al. (2012); Froehlich et al. 2023; Hartley et al. 2015; Kaplan et al. 2011; Kaslow et al. 2011; Kellen et al. 2015; Kolmes 2012; Lehavot et al. 2012; Mattison 2018; Nicholson 2011; Reamer 2013, 2017, 2018). Constant availability adds to possible boundary blurring, confusion, or crossing. Certainly, constant availability can be fruitful during crisis situations, giving clients a feeling of comfort, but it can become a slippery slope inducing boundary confusion and clients’ overdependency (Belkofer and McNutt 2011; Cooper et al. 2019; Dombo et al. 2014; Drum and Littleton 2014; Fantus and Mishna 2013; Holmes and Reid 2018; Reamer 2015, 2017).

Unclear boundaries can lead to perceiving the therapeutic relationship as more casual, intimate, and/or fluid (Baier 2019; Barnett 2019; Barsky 2017; Boddy and Dominelli 2017; Bratt 2010; Cooper et al. 2019; Drum and Littleton 2014; Fantus and Mishna 2013; Gabbard et al. 2011; Hartley et al. 2015; Kellen et al. 2015; Lannin and Scott 2013; Lehavot et al. 2012; Mattison 2018; Nicholson 2011; Reamer 2018; Smith et al. 2023). Moreover, some authors point to possible boundary violations which can lead to the exploitation of or harm to clients (Baier 2019; Barnett 2019; Bratt 2010; Drum & Littleton 2014; Gabbard et al. 2011; Jordan et al. 2014; Lannin and Scott 2013; Smith et al. 2023). From an ethical perspective, some authors argue that boundary violations are always unacceptable while boundary blurring, confusion, and crossing can be ethically acceptable depending, for example, on the needs of clients (Barnett 2019; Barsky 2017; Bratt 2010; Cooper et al. 2019; Frankish et al. 2012; Hartley et al. 2015; Jordan et al. 2014; Kaplan et al. 2011; Kolmes 2012) but not on those of professionals (Barnett 2019; Gabbard et al. 2011; Jordan et al. 2014; Lannin and Scott 2013).

Adding to an increased potential for unclear boundaries is the function of sending ‘friending’ or ‘following’ requests or the option of reacting to someone. Moreover, these functions contain the risk of potential, current, and former clients to feel rejected if professionals were to not respond, risking future support or service (Barnett 2019; Crtalic et al. 2015; Dombo et al. 2014; Kellen et al. 2015; Reamer 2013, 2015, 2017). Accepting these requests, however, can additionally open a view on the social media networks of both professionals and clients, for example by getting access to friends lists, risking confidentiality breaches (Boddy and Dominelli 2017; Crtalic et al. 2015; Jordan et al. 2014; Kaplan et al. 2011).

### Searches

Closely related to the impact of social media use on the boundaries of the therapeutic relationship is ‘searching’. Due to social media being completely integrated into people’s lives, both professionals’ and clients’ online profile pages are frequently publicly available. Hence, searches can be carried out to find their online information. Authors point out that found information, such as professionals’ or clients’ political or religious views, sexual preferences etc., can have strong effects on the therapeutic relationship (Baier 2019; Frankish et al. 2012; Gabbard et al. 2011; Jordan et al. 2014; Kaslow et al. 2011; Nicholson 2011; Reamer 2015, 2017). The literature seems to find it more permissible that clients search for their possible or current professional rather than the professional performing a search for their client. Most searches done by clients will have harmless motivations such as pure curiosity (Baier 2019; Belkofer and McNutt 2011; Lannin and Scott 2013). A small amount possibly has malicious motivations such as harassment or stalking (Baier 2019; Belkofer and McNutt 2011; Bratt 2010). Kaluzeviciute (2020) argues that some clients, especially those belonging to marginalised or vulnerable groups, can have justified reasons to do searches about their future or current professional, for example to feel safe. Moreover, professionals need to be prepared for clients divulging their searches (Dombo et al. 2014) and need to be aware that some clients will never divulge this (Kolmes 2012).

It is argued that professionals should never do searches of clients without their consent (Barsky 2017; Frankish et al. 2012; Holmes and Reid 2018; Jordan et al. 2014; Nicholson 2011). Nevertheless, in emergency situations such as a possible suicide attempt, searches with the goal of finding relevant information and without clients’ consent, could be justified. In these situations, professionals need to rely on their sound judgment (Barsky 2017; Belkofer and McNutt 2011; Cooper et al. 2019; Frankish et al. 2012; Gabbard et al. 2011; Kaplan et al. 2011; Lannin and Scott 2013; Lehavot et al. 2012; Mattison 2018; Reamer 2015, 2018). These searches are best disclosed afterwards (Cooper et al. 2019; Frankish et al. 2012; Holmes and Reid 2018; Lannin and Scott 2013; Lehavot et al. 2012; Nicholson 2011; Reamer 2017, 2018), so that the professional cannot be perceived as acting dishonestly (Cooper et al. 2019; Holmes and Reid 2018; Kaslow et al. 2011).

### Privacy, confidentiality, and trust

As many authors point out, blurred, confused, crossed, and violated boundaries of the therapeutic relationship can impact both professionals’ and clients’ privacy and confidentiality, and the trust that exists between them (Baier 2019; Boddy and Dominelli 2017; Bratt 2010; Drum and Littleton 2014; Froechlich et al. 2023; Jordan et al. 2014; Kaluzeviciute 2020; Kaplan et al. 2011; Kaslow et al. 2011; Kolmes 2012; Lehavot et al. 2012; Reamer 2013, 2015, 2017). Although some of the privacy, confidentiality, and trust issues potentially caused by social media use are similar to issues caused by face-to-face sessions or by paper use, there is a potential increase in magnitude (Barsky 2017; Boddy and Dominelli 2017; Drum and Littleton 2014; Froehlich et al. 2023). Other authors claim, on the contrary, that the use of social media could be, in some respects, more reliable than using paper documents as these can easily be misplaced, accessed by unauthorized individuals, etc. (Dombo et al. 2014; Edwards-Stewart et al. 2019; Holmes and Reid 2018; Reamer 2013, 2015, 2017).

As mentioned before, the information found on professionals or clients can have adverse effects on the therapeutic relationship. Moreover, postings done by either party can also lead to private disclosure and confidentiality breaches (Boddy and Dominelli 2017; Drum and Littleton 2014; Gabbard et al. 2011; Jordan et al. 2014; Reamer 2015, 2017, 2018). Jordan et al. (2014) note that professionals are most often mandated reporters and have the obligation to report information indicating dangerous or harmful situations.

It needs to be accounted for that postings and shared personal information via social media may be available on the internet forever (Baier 2019; Barnett 2019; Belkofer and McNutt 2011; Boddy and Dominelli 2017; Crtalic et al. 2015; Gabbard et al. 2011; Hartley et al. 2015; Kaplan et al. 2011; Nicholson 2011; Reamer 2015). As Hartley et al. (2015) state, attention needs to be paid to people’s digital footprint. This entails that information is available to individuals beyond intended audiences and can be easily pulled out of context (e.g., it can be shared or forwarded by professionals or clients, (geo-)tagged, hacked by third parties) (Baier 2019; Barnett 2019; Barsky 2017; Belkofer and McNutt 2011; Boddy and Dominelli 2017; Crtalic et al. 2015; Drum and Littleton 2014; Frankish et al. 2012; Gabbard et al. 2011; Hartley et al. 2015; Kolmes 2012; Mattison 2018).

Hence, most authors call upon professionals’ responsibility to regulate and restrict their social media profile(s). Professionals should not be naïve in thinking that the technological services they use are privacy protected (Baier 2019; Barnett 2019; Boddy and Dominelli 2017; Cooper et al. 2019; Crtalic et al. 2015; Dombo et al. 2014; Edwards-Stewart et al. 2019; Gabbard et al. 2011; Holmes and Reid 2018; Kaplan et al. 2011; Kellen et al. 2015; Lannin and Scott 2013; Mattison 2018; Reamer 2013, 2015, 2017, 2018). Some authors argue, against the idea of professionals abstaining from social media (Baier 2019), that self-disclosure over social media is unavoidable and that aiming to completely avoid self-disclosure would show professionals’ misunderstanding of the therapeutic relationship and its inherent boundaries (Barnett 2019; Barsky 2017; Bratt 2010; Crtalic et al. 2015; Dombo et al. 2014; Fantus and Mishna 2013; Frankish et al. 2012; Lannin and Scott 2013; Nicholson 2011).

Nevertheless, multiple strategies to avoid unwarranted self-disclosure and to restrict clients’ access to professionals’ social media accounts are proposed in the literature: (1) Creating separate personal and professional accounts (Barnett 2019; Crtalic et al. 2015; Dombo et al. 2014; Frankish et al. 2012; Froehlich et al. 2023; Gabbard et al. 2011; Hartley et al. 2015; Holmes and Reid 2018; Kaplan et al. 2011; Lannin and Scott 2013; Reamer 2013, 2015; Smith et al. 2023); (2) Using a pseudonym (Barnett 2019; Lannin and Scott 2013; Smith et al. 2023); (3) Using the most restrictive privacy settings (Barnett 2019; Cooper et al. 2019; Frankish et al. 2012; Froehlich et al. 2023; Gabbard et al. 2011; Kaplan et al. 2011; Lannin and Scott 2013; Reamer 2018); (4) Applying and regularly checking encryption and firewalls (Barsky 2017; Cooper et al. 2019; Crtalic et al. 2015; Froehlich et al. 2023; Jordan et al. 2014; Kaplan et al. 2011; Mattison 2018; Nicholson 2011; Reamer 2013, 2015, 2018); (5) Doing self-searches to see what information is available and act upon it accordingly (Belkofer and McNutt 2011; Bratt 2010; Dombo et al. 2014; Frankish et al. 2012; Gabbard et al. 2011; Kaslow et al. 2011; Kolmes 2012; Lannin and Scott 2013). Although many of these strategies are the responsibility of professionals, it is also clients’ responsibility to ensure that their own privacy, and professionals’ privacy, is guaranteed as best as possible. Professionals need to inform and educate clients about the different privacy, confidentiality, and trust issues related to the use of social media (Belkofer and McNutt 2011; Cooper et al. 2019; Crtalic et al. 2015; Edwards-Stewart et al. 2019; Fantus and Mishna 2013; Froehlich et al. 2023; Kellen et al. 2015; Reamer 2018).

Using social media also creates new ways of impacting professionals’ and clients’ privacy and confidentiality. Online data can be accessed by third parties (e.g., hacking) (Barsky 2017; Cooper et al. 2019; Crtalic et al. 2015; Dombo et al. 2014; Jordan et al. 2014; Mattison 2018; Reamer 2013, 2015, 2018) or could be unintentionally sent to the wrong person (Reamer 2018). Different data servers (e.g., computers, phones, etc.) may be stolen, misplaced, or physically accessed by unauthorized parties (Barsky 2017; Cooper et al. 2019; Edwards-Stewart 2019; Jordan et al. 2014; Nicholson 2011; Reamer 2018).

### Documentation and records

Besides the difficulties that using social media in social care can cause in relation to privacy, confidentiality, and trust, it also causes new challenges for service documentation and client records. Proper documentation and records are necessary for assessing, planning, and delivering services, or to be accountable to clients, professional agencies, other providers, and courts. Moreover, documentation and records are used to ensure coordination and continuation of services (Barsky 2017; Frankish et al. 2012; Reamer 2015, 2017). Hence, strict protocols about which and how documentation and records will be stored and destroyed need to be developed and followed so that good quality services and the continuation thereof are guaranteed (Bratt 2010; Frankish et al. 2012; Holmes and Reid 2018; Nicholson 2011; Reamer 2013, 2015, 2017). Different social media usages (e.g., video-calling, messaging, carried out searches) need to be documented and included in client records (Drum and Littleton 2014; Reamer 2015, 2017, 2018).

The need to secure the confidentiality of documentation and records in line with relevant laws and regulations is emphasized by multiple authors. Moreover, professionals need to be familiar with exceptions to this confidentiality (Drum and Littleton 2014; Frankish et al. 2012; Holmes and Reid 2018; Mattison 2018; Nicholson 2011; Reamer 2015, 2017, 2018) such as when professionals are subpoenaed or receive court orders to release stored information (Reamer 2015, 2017, 2018). Reamer (2015, 2017) also argues that clients and professional colleagues need to have reasonable and appropriate access to both electronic and physical documentation and records.

### Competency and client suitability

As with any other social care practice, social media use demands that professionals are competent in using it. Competency at least entails that professionals know the benefits and the risks of using social media, how communications develop over social media channels (e.g., use of emojis, graphic interchange formats (GIFs), abbreviations), how to interpret and handle clients’ postings, how boundaries and trust are affected, and how to set privacy settings (Belkofer and McNutt 2011; Boddy and Dominelli 2017; Bratt 2010; Cooper et al. 2019; Crtalic et al. 2015; Dombo et al. 2014; Drum and Littleton 2014; Edwards-Stewart et al. 2019; Fantus and Mishna 2013; Frankish et al. 2012; Froehlich et al. 2023; Hartley et al. 2015; Holmes and Reid 2018; Jordan et al. 2014; Kellen et al. 2015; Kolmes 2012; Mattison 2018; Nicholson 2011; Reamer 2013, 2017, 2018; Smith et al. 2023). Clients too need a certain level of competency as a good understanding of social media and its use will protect both themselves and professionals. Moreover, clients need to create a realistic expectation about the therapeutic relationship and understand how not to use social media in it (Barnett 2019; Barsky 2017; Belkofer and McNutt 2011; Cooper et al. 2019; Crtalic et al. 2015; Edwards-Stewart et al. 2019; Fantus and Mishna 2013; Hartley et al. 2015; Holmes and Reid 2018; Kellen et al. 2015; Reamer 2018).

Some authors argue that while developing competency for themselves and their clients, professionals need to remain aware of their clients’ cultural sensitivities and unique needs (e.g., linguistic sensitivities, social and economic needs) (Barsky 2017; Cooper et al. 2019; Drum and Littleton 2014; Kolmes 2012; Reamer 2018). Finally, professionals should have a minimal standard of technical mastery. As social media can only exist through certain software programs (e.g., web browsers, apps) and hardware installations (e.g., computer, smartphone), professionals relying on social media should be able to ensure that these programs and installations work properly (Cooper et al. 2019; Crtalic et al. 2015; Dombo et al. 2014; Edwars-Stewart et al. 2019; Holmes and Reid 2018; Jordan et al. 2014; Reamer 2015, 2018; Smith et al. 2023).

To uphold a sufficient level of competency, it is necessary that professionals review pertinent research and practice literature on social media use (Fantus Mishna 2013; Kaslow et al. 2011; Kellen et al. 2015; Reamer 2013, 2015, 2017, 2018). Reamer (2013) calls to check the quality of available research, hinting that the current quality does not always meet the highest standards. Moreover, if necessary, professionals need to seek training and education on social media counselling to deal with possible challenges (e.g., missing communication cues) (Bratt 2010; Cooper et al. 2019; Dombo et al. 2014; Fantus and Mishna 2013; Froehlich et al. 2023; Gabbard et al. 2011; Holmes and Reid 2018; Jordan et al. 2014; Kaslow et al. 2011; Kolmes 2012; Lannin and Scott 2013; Mattison 2018; Reamer 2015, 2017, 2018; Smith et al. 2023).

Besides being able to handle social media according to an appropriate standard of mastery, professionals’ competency also entails being able to assess whether clients are suited to be treated via practices relying on social media (Barsky 2017; Cooper et al. 2019; Dombo et al. 2014; Drum and Littleton 2014; Froehlich et al. 2023; Jordan et al. 2014; Mattison 2018; Reamer 2017). Some clients and potential clients with specific problems might not be served with social media use (Reamer 2013) and could experience adverse outcomes: those with thoughts of hurting themselves or others; those with a history of suicidal, violent, abusive tendencies; those with delusions or hallucinations; those being active alcohol or drug abusers (Dombo et al. 2014); those with compulsive behaviors towards technology (e.g., need of being constantly online and available) (Reamer 2018); and those with a history of disturbed interpersonal boundaries (Cooper et al. 2019; Drum and Littleton 2014).

### Consultation and referral

If clients are unsuited to be involved in social media care practices, professionals need to provide them with high-quality alternatives (Barsky 2017) or refer them to colleagues (Barsky 2017; Cooper et al. 2019; Dombo et al. 2014; Jordan et al. 2014; Reamer 2017). Additionally, clients need to know how to react if they find themselves in an emergency situation or if there is a technical malfunction, making social media use impossible. They need to know how to locate and access emergency counselling and other supportive services (e.g., suicide hotlines) (Cooper et al. 2019; Crtalic et al. 2015; Dombo et al. 2014; Frankish et al. 2012; Froehlich et al. 2023; Jordan et al. 2014; Kaslow et al. 2011; Kellen et al. 2015; Reamer 2013, 2018). As Reamer (2013) argues, establishing this information and these alternative counselling channels is itself impeded by social media use as the chance exists that professionals and clients do not meet each other in person, that professionals and clients do not live in the same community, or because professionals do not have a professional network in their clients’ community.

With social media, the risk of abandoning a given social care service increases. Clients can suddenly disappear online or stop responding to professionals’ attempts to communicate (Boddy and Dominelli 2017; Reamer 2013). But also on the part of professionals, services can be interrupted by technical failure or by being unable to respond in a timely manner (Crtalic et al. 2015; Reamer 2013). Hence, reasonable arrangements to continue services need to be guaranteed (Reamer 2013).

### Identity and image

Social media use in social care makes it increasingly difficult to confirm the identity of professionals and clients alike. In the case of confirming professionals’ identity, clients can ask for their licenses or certificates at the beginning of each social media consultation session (Kaplan et al. 2011; Kaslow et al. 2011). Authors discuss the possible risk of clients’ identity theft or fraud (Cooper et al. 2019; Crtalic et al. 2015; Holmes and Reid 2018; Kaplan et al. 2011; Mattison 2018; Reamer 2013, 2017, 2018). Also, clients can more easily present themselves differently online than in real life or could make use of a pseudonym. This urges both professionals and clients not to take online information and profiles at face-value (Boddy and Dominelli 2017; Bratt 2010; Cooper et al. 2019; Kaluzeviciute 2020; Kaslow et al. 2011; Kolmes 2012; Lehavot et al. 2012). At least one strategy to deal with this insecurity is for professionals and clients to agree on a shared ‘password’ by which they can identify each other (Crtalic et al. 2015; Kaplan et al. 2011). Additionally, some authors also point out the difficulty of assessing clients’ age via social media (Cooper et al. 2019; Holmes and Reid 2018; Reamer 2017).

Besides the topic of identity, some authors point out that professionals need to keep in mind which online image they create. For many, it is the professionals’ responsibility to make sure that their online activity is in accordance with the respectful and appropriate representation of their profession (Baier 2019; Barsky 2017; Belkofer and McNutt 2011; Boddy and Dominelli 2017; Dombo et al. 2014; Drum and Littleton 2014; Frankish et al. 2012; Froehlich et al. 2023; Gabbard et al. 2011; Kaluzeviciute 2020; Kellen et al. 2015; Smith et al. 2023). Reamer (2013, 2015, 2018) and Smith et al. (2023) argue that conflicts of interest can occur as professionals making use of certain social media platforms or ‘liking’, ‘sharing’ or ‘following’ social media pages of specific private services, could give the impression that they endorse the companies behind them.

### Informed consent

The bigger part of the included articles underlines that a way to deal with all described ethical tensions related to social media use in social care is for professionals to detail them in protocols and policies together with their stance towards each of these issues. These protocols and policies need to be discussed with clients before the use of social media (Barnett 2019; Barsky 2017; Belkhofer and McNutt 2011; Cooper et al. 2019; Crtalic et al. 2015; Dombo et al. 2014; Drum and Littleton 2014; Edwards-Stewart et al. 2019; Fantus and Mishna 2013; Froehlich et al. 2023; Hartley et al. 2015; Holmes and Reid 2018; Jordan et al. 2014; Kaplan et al. 2011; Kaslow et al. 2011; Kellen et al. 2015; Kolmes 2012; Lannin and Scott 2013; Lehavot et al. 2012; Mattison 2018; Nicholson 2011; Reamer 2015, 2017; Smith et al. 2023). Professionals could revert to in-person consultations if these protocols and policies are not followed (Crtalic et al. 2015; Mattison 2018).

Nevertheless, professionals must ensure clients understand what it means to receive services via the use of social media, with all its benefits and risks, and related policies and protocols (Barsky 2017; Belkofer and McNutt 2011; Cooper et al. 2019; Edwards-Stewart et al. 2019; Fantus and Mishna 2013; Frankish et al. 2012; Froehlich et al. 2023; Hartley et al. 2015; Kaplan et al. 2011; Kellen et al. 2015; Lannin and Scott 2013; Mattison 2018; Reamer 2013, 2015, 2017; Smith et al. 2023). Moreover, professionals need to assess if clients are mentally capable to give their consent for using or not using social media in their care which can be daunting when professionals and clients would only meet via social media (Barsky 2017; Cooper et al. 2019; Frankish et al. 2012; Kaplan et al. 2011; Reamer 2013, 2015, 2017).

Clients need to be autonomous when giving their consent, meaning that they cannot be forced, manipulated, etc. into consent (Barsky 2017; Bratt 2010; Hartley et al. 2015; Kaslow et al. 2011; Lehavot et al. 2012; Reamer 2013). Nevertheless, Boddy and Dominelli (2017) indicate that the continuous availability of data on social media undermines any form of genuine consent by the client**.** Holmes and Reid (2018) and Reamer (2013, 2015, 2017) indicate the difficulty of professionals providing services to minors as laws differ regarding the necessary parental consent. Finally, when considering clients’ autonomy, social media protocols and policies can lay the basis for a therapeutic relationship and social media use which is grounded in trust between professionals and clients (Crtalic et al. 2015; Kaplan et al. 2011; Kolmes 2012; Mattison 2018).

### Outliers

Boddy and Dominelli (2017) and Steiner (2021) explicitly go beyond focusing on the therapeutic relationship and beyond the identified nine themes. Boddy and Dominelli (2017) address the macro-level socio-political context in which this relationship develops. They analyze this context for relevant ethical implications of the pertinent social background structure, which they see as neoliberal and consisting of the tendency to commodify care relations, and consider the ethically relevant characteristics of online spaces created by social media. They do, however, also address some of the nine identified themes. The publication by Steiner (2021), by contrast, lends itself less to be analyzed within the framework of these themes. It urges social work professionals to adopt an ethics of responsibility, i.e. to strive to actively shape digital technologies, construed as formable and fundamentally open, according to social work values (e.g., social equality, power distributions, importance of relationships, inclusion).

## Discussion

Increasingly, digital technologies such as social media are pervading different settings of human life. This upsurge is joined with different benefits and vulnerabilities. In this systematic review, we laid bare these benefits and vulnerabilities related to the use of social media in social care by critically assessing the ethical arguments used in the debate on this use. Thirty-three argument-based ethics articles about social media use in social care were included and analysed. Inspired by the PRISMA statement for systematic reviews (Liberati et al. 2009), we first developed two conceptual-ethical research questions that guided the development of this review, focussing on reaching an exhaustive overview and in-depth insight into the argumentation used in the debate on using social media in social care. Second, a detailed search string was developed and applied in four electronic databases to identify possibly relevant literature. Third, pre-defined inclusion and exclusion criteria were applied to identify peer-reviewed articles eligible to be included. Snowballing was applied on included articles’ reference lists to identify literature that may have been overlooked. Fourth, inspired by the QUAGOL (Dierckx de Casterlé et al. 2012) and with a modified version of Vandemeulebroucke et al. (2022c) data-extraction tool, the content of the different articles was analysed.

In this review we identified nine analytical themes based on the content of the included articles, except for two outliers (Boddy and Dominelli 2017; Steiner 2021). As such, these themes govern the current debate on the ethics of the use of social media in social care: (1) Benefits of social media; (2) Relations, limits, and boundaries; (3) Searches (4) Privacy, confidentiality, and trust; (5) Documentation and records; (6) Competency and client suitability; (7) Consultation and referral; (8) Informed consent; (9) Identity and image. This systematic review makes evident that the topic of the ethics of the use of social media in social care is a well-researched one, but mainly by researchers from within social care disciplines, and thus outside of the philosophical-ethical field. As such, the current ethics debate mainly remains on a practical level and is in need of a deeper conceptual analysis. Moreover, this focus on the ethics of the use of social media stands in sharp contrast with the indicated lack of research on the actual use of social media in social care settings (Chan 2016; Cooner et al. 2020).

### The ethics of social media use in social care

This systematic review shows that the current ethics of social media use in social care almost exclusively focusses on the particular therapeutic relationship between professionals and clients. Indeed, the nine themes described refer to this relationship and how it is impacted by social media technology. Nevertheless, as it now stands, the literature seems to perceive the ethical impact of social media use in social care, and explicitly the therapeutic relationship, as either positive or negative. The image of social media exhibiting this positive and negative side simultaneously shines through only tentatively.

Relying on postphenomenology, we argue that this two-sidedness is essential to social media technologies and entails more than solely its ethics. Putting the therapeutic relationship at the centre, we ask how social media mediates this relationship, rather than how social media impacts it. Following Kiran (2015), we discern an inherent two-sided dynamic to this mediation along four dimensions, by which we can describe social media as simultaneously revealing and concealing aspects of reality (ontological), magnifying and reducing avenues to knowledge of that reality (epistemological), enabling and constraining possibilities for action (practical), and involving professionals and clients with or alienating them from the therapeutic relationship and ideas of its inherent good (ethical). While the majority of the literature finds both positive and negative effects of social media on the therapeutic relationship, it fails to establish a common cause or systematic link between these effects. Conceptualizing the therapeutic relationship as fundamentally mediated by two-sided dynamics instead of as essentially static proves insightful for addressing and anticipating effects.

The nine themes established by this review reveal different ways of how these different two-sided dynamics can be balanced out to increase the potential positive effects of social media usage while minimizing the negative. These themes indicate that the current literature is inspired by the principles of biomedical ethics (Beachamp and Childress 2019) to guide this mediating nature of social media: beneficence and non-maleficence (themes: (1) Benefits of social media; (2) Relations, limits, and boundaries; (3) Searches (4) Privacy, confidentiality, and trust; (5) Documentation and records; (6) Competency and client suitability; (7) Consultation and referral), respect for autonomy (themes: (8) Informed consent; and (9) Identity and image), and justice (theme: (1) Benefits of social media).

This sole focus on the therapeutic relationship and this inspiration by biomedical ethics indicate that the current literature is guided by the question of how a careful use of social media can be guaranteed to increase its potential positive effects. This ethics of carefulness (Bolte et al. 2022; Vandemeulebroucke et al. 2022a; b) takes social media technologies and their trajectory for granted and as such works from inside the technical paradigm. This paradigm thus proposes ethical principles, here the principles of biomedical ethics, that function as criteria along which the careful social media use can be evaluated. Notably, Steiner (2021) opposes this view by calling to actively shape digital technologies, i.e., social media, according to social work values.

Because it takes social media for granted, the ethics of carefulness focusses on the digital dimension of social media and its digital and physical effects. Moreover, it makes social media use in social care the responsibility of individual professionals and clients. Except for Boddy and Dominelli (2017) and Steiner (2021), the socio-political determinants that led to the use of social media are left out of consideration. Consequently, the literature largely overlooks that each care practice is always a “caring with” (Tronto 2013; Vandemeulebroucke et al. 2022a). Translated into an ethical approach, “caring with” contains four relevant and interrelated levels which are ethically impacted by the use of social media in social care (Vandemeulebroucke 2022): (1) Individual level—This level contains the specific ethical impacts social media use has on the particular therapeutic relationship. Most of the included literature deals with this level; (2) Organizational level—Each social care practice, also those relying on social media, is carried out in an organizational setting (e.g., family, facilities, organizations). Ethical impacts here relate to, for example, what an organization’s motivations are for using social media (e.g., providing better care, saving costs) or how social media usage relates to organizations’ identity and deontological codes; (3) Societal level—From a societal perspective, questions arise about what social media use tells us about how we conceive of social media care practices from a societal perspective. Is it indeed the case, as Boddy and Dominelli (2017) hint at, that the use of social media is an expression of and leads to a further commodification of care? Or does the use of social media advance increasingly accessible social care, as many authors suggest? (4) Global level—It needs to be recognized that social media technologies consist of more than a merely digital dimension. Social media technologies need to be considered as ‘world objects’ affecting the world in its entirety (Feenberg 2017), and thus embodying different networks of technical-environmental-human relationships. This conception recognizes that social media have a material dimension besides their digital one, considering, for example, the different hardware technologies (e.g., servers, computers, smartphones) needed to produce social media. Hence, ethical questions arise about what the climate and environmental impact is of using social media in social care (Hischier et al. 2015) or how to address bad labour conditions of people who have to mine the natural resources (e.g., gold, copper, tungsten) and develop technical resources (e.g., computer chips, electric wiring) required for the production of the hardware (Bolger et al. 2021).

Together, these four levels of different ethical impacts of social media use lead to a perspective of an ethics of desirability (Bolte et al. 2022; Vandemeulebroucke et al. 2022a; c). From this perspective, we ask: What is the meaning of social media and for whom is it desirable, on which of the four identified levels of analysis is it desirable? Markedly, what counts as ethically desirable and meaningful on one level, does not necessarily do so on other levels. It is only through an integrated view that the full ethical impact of social media use in social care can be understood. Inspired by a question methodology (Hofmann 2005, 2017), Table 6 gives a rudimentary reflection guide which can inspire critical reflection on the use of social media in social care from the different levels of ethical impact: individual, organisational, societal, and global.

**Table 6:** Guiding reflection questions on the use of social media in social care according to four levels of ethical consideration

Guiding reflection questions
Global level
∙ How does the use of social media relate to global issues such as care as a basic human right, global care inequalities,…? ∙ How does the use of social media relate to global issues such as energy consumption, climate change, and environmental issues,…? ∙ How does the use of social media relate to a global market governed by multinational companies? ∙ Why do we (not) need to take these and other global issues into account in our assessment of social media use in social care? ∙…
Societal level
∙ What does the use of social media show us about society’s current conception of social care? How will the use of social media possibly impact society’s conception of social care? ∙ Will the use of social media increase the accessibility of social care practices? In which way will this use do this? In which way will this use obstruct the accessibility of social care practices? ∙ How will the use of social media meet existing social care inequalities and/or discrimination? Does the possibility exist that the use of social media in social care will create new inequalities and/or discrimination? ∙ How do we account for bringing private companies into existing social care settings? Do we, as a society, have the necessary ethical and legal frameworks? If not, how do we deal with this lack of guidance? ∙…
Organisational level
∙ What motivates our organisation to implement the use of social media? What justifies these motivations? ∙ How does the use of social media relate to the organisation’s current social care practices? Will this use complement or replace existing practices? How is this use justified? ∙ How does the use of social media fit with our organisation’s deontological code and broader ethical guidelines (e.g., social workers associations, psychologists associations)? ∙ Does everyone in the organisation need to use social media? Why do they? Why do they not? How is it decided who will use social media? ∙…
Individual level
∙ How can we expect that the use of social media in social care will mediate particular therapeutic relations? How do we ensure that relations between professionals and clients remain within the boundaries of therapeutic relevance? ∙ When is it appropriate to search for a client? What justifies this search? How can professionals and/or clients disclose searches done to one another in a safe and trustworthy manner? When do these searches need to be disclosed? Why do searches need to be disclosed? Why do they not need to be disclosed? ∙ How will professionals and/or clients be informed about the highest privacy settings on their social media accounts when these are used to establish interaction? How will professionals and/or clients be informed that interactions via social media are the property of private companies? Do professionals need to create a professional social media account? Why do they? Why do they not? ∙ What measures will be implemented to assure that social media interactions between professionals and clients are documented to the highest standards? How will it be ensured that these records are accessible to colleagues under applicable deontological and legal frameworks (e.g., when a professional retires, when documentation is subpoenaed)? ∙ How do we determine if professionals and/or clients are competent to interact with each other via social media? ∙ How do we assess clients’ suitability to be involved in social media care practices? Which profiles of clients could be involved in social media care practices and which ones should not? What motivates this inclusion and exclusion? ∙ How will the advantages and disadvantages of the use of social media be disclosed? What are the implications of social media use in particular therapeutic relations? In what manner will this information be relayed in an approachable way? Are clients capable of understanding this information and give their informed consent to the use of social media in their care? How are vulnerable clients approached and how will their informed consent be obtained? ∙ How do we assure that professionals and/or clients that present to them over social media are indeed the persons who they say they are? Should a face-to-face interaction between professionals and clients precede possible social media interaction? Why so, why not? ∙…

The ellipses “…” indicate that these questions are not exhaustive and can be modified or complemented according to any particular social care practice and situation

As such, the ethics of desirability is a perspective that forces us to look beyond the current technical paradigm and beyond the confounds of the local social care context and relationship, and places social care, with and without social media alike, in continuously enlarging technical-environmental-human contexts, thus ensuring care for all everywhere.

### Strengths and limitation

This systematic review’s search and selection process was inspired by the PRISMA-statement (Liberati et al. 2009) and followed clear predefined in- and exclusion criteria enabling the identification of the included 33 peer-reviewed articles. The analysis process was inspired by the first five preparatory phases of the QUAGOL (Dierckx de Casterlé et al. 2012) and relied on a modified version of the analysis tool developed by (Vandemeulebroucke et al. 2022c) guaranteeing continuity in the analysis of each individual article. Despite these strengths, certain limitations need to be pointed out.

The search was limited to articles published from 2010 onwards. Hence, the possibility exists that this review overlooked relevant articles. Nevertheless, as this review’s focus lies on being exhaustive in relation to ethical argumentation and not articles per se, and because many well-known social media platforms have found global acceptance in the last decade or so, we contend that this time limitation is justified. It is surprising that the included articles discussed examples of which some can be considered old (e.g., Myspace), well-established (e.g., Facebook), or even platforms that commonly would not always be perceived as social media (e.g., Wikipedia), while neglecting a new generation of platforms (e.g., TikTok). This insight is congruent with Chan’s (2016) scoping review on social media use in social work settings in which he noticed conceptual unclarity about what social media is and which platforms it entails.

Included articles came from a diverse field of scholarly literature, including psychology, social work, rehabilitation, psychiatry, art therapy and, family, couple, and marriage therapy. Despite the thoroughness of the methodology employed, all included articles stem from research being done in western countries. Moreover, all but one originated in English-speaking countries. This global north focus can be explained by considering which electronic databases were consulted, the fact that the search string was developed in English, and the authors’ language restrictions. Nevertheless, it could also point to an unequal distribution of technology or unequal access to healthcare services between countries. It could also be that there remains a social stigma on different forms of social care (e.g., mental health care) in different regions which is reflected in current research. Taking into account that social media is a world-object, including non-western ethical traditions, theories, and frameworks in future research would create necessary input to develop more fine-grained ethical considerations. Hence, we conceive of the current review as only one part of a bigger story about the use of social media in social care.

## Conclusion

There is an increasing ethical awareness of the possibilities that come with social media use in social care practices like social work, psychology, rehabilitation, etc. This systematic review identified nine themes of which each related to possible ethical vulnerabilities introduced in the therapeutical relationship between professionals and clients by the use of social media. Although this focus on the particular therapeutic relationship is understandable and necessary, as it should lead to a careful use of social media, it is not the whole story. This review made clear that the exclusive focus on the therapeutic relationship obscures the organizational, societal, and global levels which are impacted by this relationship and social media. By taking these broader contexts of the ethical assessments of social media in social care practices into account, the ethical lens shifts from an ethics of carefulness to an ethics of desirability. Further research should place itself into the tension between these two ethical perspectives as to create in-depth insight into all the ethical aspects of social media use in social care, to consequently establish an integrated ethical account of this practice, one that is in line with those social care practices we all desire.

## Supplementary Information

Below is the link to the electronic supplementary material.

Supplementary file1 (DOCX 78 KB)

## Data Availability

Online additional data.
