# Supplementary material for: The use of social media in social care: a systematic review of the argument-based ethics literature
Source: Med Health Care Philos. 2025 May 3;28(3):639–65. doi: 10.1007/s11019-025-10269-4 (PMC12380649; doi:10.1007/s11019-025-10269-4)
Supplement: Supplementary file 1 — Supplementary file1 (DOCX 78 KB) [file 11019_2025_10269_MOESM1_ESM.docx]

### **Additional files**

**Additional file 1 – Final Version Data Extraction Tool**

**[Referenced article]**

Research Questions:

What are the arguments used in the ethical debate on the use of social media in welfare settings?

What are the ethical concepts grounding these arguments?

Characteristics of publication

**Language:**

**First author’s publication country:**

**Profession/Research discipline first author:**

**Research field:**

**Focus of Journal:**

**Year of publication:**

Arguments and ethical concepts

**Which social media**:

1. Benefits social media
2. Risks social media
3. Relations, limits and boundaries
4. Privacy, confidentiality and trust
5. Documentation and records
6. Competency
7. Problematic behaviour and client suitability
8. Consultation and referral
9. Informed consent
10. Identity and image
11. Others

**Ethical approach:**

**Additional file 2 – Example of conceptual scheme**

**Reamer, F. G. (2013). Social Work in a Digital Age: Ethical and Risk Management Challenges. *Social Work, 58*(2), 163-172. doi:10.1093/sw/swt003**

Research Questions:

What are the arguments used in the ethical debate on the use of social media in welfare settings?

What are the ethical concepts grounding these arguments?

Characteristics of publication

**Language:** English

**First author’s publication country:** United States of America

**Profession/Research discipline first author:** Social Work **ethics**

**Research field:** Social work

**Focus of Journal:** Social work

**Year of publication:** 2013

Arguments and ethical concepts

**Which social media**: social media/SNS as part of digital technology

1. Benefits social media

Increased accessibility

- People with anxiety/extreme shyness
- People who are disabled, live remotely
- Need for help after hours, when in a crisis

1. Risks social media

- Missing visual and nonverbal cues
- Risk of communication misunderstandings
- Some people with specific difficulties not served with electronic help
- Technology failure, confidentiality breaches
- Loss of reasonable expectations of a turnaround time for responses
  - May lead to misunderstanding & conflict
- Risk of violating state laws
- Risk of encountering identity fraud

1. Relations, limits, and boundaries

Social networking sites -> friend requests of clients and former clients

- Boundary confusion
- Comprise clients’ privacy & confidentiality
- Comprise professional’s privacy & confidentiality
  - Complex transference and countertransference issues in professional – client relationship
  - Create personal and professional page
- Not accepting friend requests -> lead to a feeling of rejection

Conflict of interest

- Making use of specific site/service -> give impression that professional endorses activities of company/provider (e.g. counselling site sponsors)

1. Privacy, confidentiality and trust

Social media/social networking sites -> new layer of challenging issues

- E.g. makes use of sophisticated encryption technology to avoid unauthorized 3^rd^ parties to access data and comply with it

Health Insurance Portability and Accountability Act (HIPAA)

- Current technology > traditional paper documents
  - Some technology is better protected than others
  - Ethical burden on professional to ensure trustworthy encryption

1. Documentation and records

Good encryption technologies

Need for strict protocols to ensure clinically relevant exchanges are properly documented in case records

1. Competency

Need to meet minimal standards of competence

Review pertinent research & practice literature

- Become familiar with rapidly emerging ethical standards
- Assess the quality of research -> best quality = randomize control trials

1. Consultation and referral

Need for steps to ensure clients are familiar with info they would need to locate and access emergency, counselling, case management, and other supportive services

- Becomes difficult/impossible when:
  - Professional does not meet the client in person
  - Professional and client do not live in the same community
  - Professional does not have a professional relationship with other service providers
- Lead to inadequate coordination of services and incomplete/inaccurate clinical assessments

1. Informed consent and policy

Electronic devices -> duty to ensure clients understand nature of services and potential benefits & risks

- Can be difficult when client & professional never meet in real life

Difficulty of minors -> state laws differ regarding parental consent

Standards of consent to be considered:

- Avoid coercion, undue influence, pressure
- Client must be mentally capable to give consent
  - Need to assess capacities and current circumstances
    - Can be difficult when not meeting in person -> confirming identity and age
- Online consent forms and procedures must be valid

Need for social media policy (Kolmes 2010) -> risk management strategy

1. Others
2. Termination/interruption of services

Risk of abandonment

- Need to make reasonable arrangements for continuation of services

Online/electronic services terminated for variety of reasons

- Clients terminate services abruptly -> disappear, do not respond
- Professional terminates services -> technical failure, failing to respond in a timely manner

1. Research evidence

Very little high quality, compelling research demonstrating evidence of effectiveness

**Ethical approach:** Systematic application of Standards, Codes of Ethics

- International society for mental health online (UK-based)
- Association for Counseling and Therapy Online
- American Telemedicine Association
- National Association of Social Worker’s Code of Ethics

**Additional file 3 - Overarching conceptual scheme**

| **Included Themes** | | | | | | | | |
| --- | --- | --- | --- | --- | --- | --- | --- | --- |
| **Themes** | ***Subthemes*** | **Description** | | | | | | **References** |
| **Benefits social media** | *Increased accessibility* | People with anxiety, extreme shyness, agoraphobia -> increased feelings of emotional safety/comfort (Cooper et al. 2019; Crtalic et al. 2015; Dombo et al. 2014; Holmes & Reid 2018; Mattison 2018;); flexibility and spontaneity (Frankish et al. 2012) | | | | | | Cooper et al. 2019; Crtalic et al. 2015; Dombo et al. 2014; Fantus & Mishna 2013; Frankish et al. 2013; Froehlich et al. 2023; Hartley et al. 2015; Holmes & Reid 2018; Kellen et al. 2015; Reamer 2013; Reamer 2015 |
|  |  | People who are disabled, live remotely, who are ill (undeserved groups of people) (reduced/eliminated transportation barriers) | | | | | | Barsky 2017; Cooper et al. 2019; Crtalic et al. 2015; Dombo et al. 2014; Fantus & Mishna; Hartley et al. 2015; 2013; Reamer 2013; Reamer 2017; Reamer 2018 |
|  |  | 24/7/365 availability -> Need for help after hours, during a crisis (convenient scheduling) | | | | | | Cooper et al. 2019; Lehavot et al. 2012; Reamer 2013; Reamer 2015; Reamer 2017; Reamer 2018 |
|  |  | Many (in the US) have a smartphone, even in a weaker socio-economic position (= underserved populations Jordan et al. 2014) | | | | | | Edwards-Stewart et al. 2019; Jordan et al. 2014 |
|  | *Increased therapeutic value* | Empower clients, client preference | | | | | | Cooper et al. 2019; Froehlich et al. 2023; Hartley et al. 2015; Kellen et al. 2015; Mattison 2018; Reamer 2015; Reamer 2017 |
|  |  |  | Increased privacy -> individuals who do not want to be recognized in waiting room | | | | | Mattison 2018; Reamer 2015; Reamer 2017 |
|  |  |  | Less anxiety during self-disclosure -> increase feelings of emotional safety | | | | | Cooper et al. 2019; Dombo et al. 2014; Fantus & Mishna 2013; Kellen et al. 2015 |
|  |  |  | Avoid public stigma and consequences of strict social norms | | | | | Hartley et al. 2015; Kellen et al. 2015 |
|  | *Contact* | More formal contact -> positive and supportive messages | | | | | | Hartley et al. 2015; Reamer 2015; Reamer 2018 |
|  | *Effectiveness* | Cost-effectiveness | | | | | | Cooper et al. 2019; Crtalic et al. 2015; Jordan et al. 2014; Kellen et al. 2015; Reamer 2018 |
| **Risks social media** |  | Compromises professionals’ ability to comply with core ethical values/standards (e.g. privacy, informed consent, confidentiality, professional boundaries, competent practice, termination of practice etc.) | | | | | | Dombo et al. 2014; Froehlich et al. 2023 ; Reamer 2015; Reamer 2018 |
|  |  | Higher costs of technology -> unequal access | | | | | | Hartley et al. 2015; Holmes & Reid 2018; Mattison 2018; Reamer 2018 |
| **Self-disclosure, friending, relations, limits, and boundaries** | *Boundaries* | Rules that govern therapeutic relationships and which help to differentiate it from business/social relationship -> makes therapeutic relationship flourish | | | | | | Drum & Littleton 2014; Frankish et al. 2012 |
|  |  | Professional relationship = time-limited + context depending + certain anonymity -> no free-standing relationship | | | | | | Crtalic et al. 2015; Drum; & Littleton 2014; Kaluzeviciute 2020 |
|  | *Real – virtual therapeutic relationship* | Offline clinical encounter is impeded, modified and influenced by something happening in the online world | | | | | | Kaluzeviciute 2020 |
|  | *Multiple relations – blurred boundaries - Friending* | Conceive therapeutic relationship as more casual/social/personal/intimate/fluid (e.g. language use Cooper et al. 2019) | | | | | | Baier 2019; Barnett 2019; Barsky 2017; Bratt 2010; Cooper et al. 2019; Drum; & Littleton 2014; Fantus & Mishna 2013; Gabbard et al. 2011; Hartley et al. 2015; Kellen et al. 2015; Lannin & Scott 2013; Lehavot et al. 2012; Mattison 2018; Nicholson 2011; Reamer 2018; Smith et al. 2023 |
|  |  | Boundary blurring/confusion/crossings with clients and former clients | | | | | | Barsky 2017; Bratt 2010; Crtalic et al. 2015; Drum & Littleton 2014; Frankish et al. 2012; Froehlich et al. 2023; Hartley et al. 2015; Kaplan et al. 2011; Kaslow et al. 2011;Kellen et al. 2015; Kolmes 2012; Lehavot et al. 2012; Mattison 2018; Nicholson 2011; Reamer 2013; Reamer 2017; Reamer 2018 |
|  |  | Boundary violations   - Inappropriate, unacceptable unethical   Significant for exploitation of and **harm** to client | | | | | | Baier 2019; Barnett 2019; Bratt 2010; Drum; & Littleton 2014; Gabbard et al. 2011; Jordan et al. 2014; Lannin & Scott 2013; Smith et al. 2023 |
|  |  | Can lead to professional’s impaired objectivity and judgement (transference problem) | | | | | | Barnett 2019; Bratt 2010; Froehlich et al. 2023; Jordan et al. 2014; Kaslow et al. 2011; Kaluzeviciute 2020; Kellen et al. 2015; Lannin & Scott 2013; Smith et al. 2023 |
|  |  | Should be resolved immediately | | | | | | Bratt 2010; Smith et al. 2023 |
|  |  | If non-professional relation is formed -> needs to be documented and informed consent needs to be obtained | | | | | | Crtalic et al. 2015 |
|  | *Overlapping networks* | Patient and professional may have overlapping network (e.g. member of same online group) -> Avoid conflict of interest | | | | | | Barsky 2017; Jordan et al. 2014; Kolmes 2012; Nicholson 2011; Reamer 2018 |
|  | *(Non-verbal) communication as difficult* | Risk of communication misunderstandings | | | | | | Cooper et al. 2019; Dombo et al. 2014; Jordan et al. 2014; Kolmes 2012; Mattison 2018; Reamer 2013 |
|  |  |  | | Client/professional can become confused about a send message/can perceive different meaning in a message than intended one -> can cause conflict in therapeutic/counselling relationship | | | | Crtalic et al. 2015; Drum; & Littleton 2014; Jordan et al. 2014 |
|  |  | Missing clinical cues (e.g. visual and nonverbal cues) | | | | | | Dombo et al. 2014; Mattison 2018; Reamer 2013; Reamer 2015 |
|  | *Negative Consequences multiple relations – blurred boundaries – self-disclosures – Friending* | Not accepting friends requests/unfried/not reacting (Dombo et al. 2014) -> risk of deep feeling of rejection, risks client not returning to treatment in the future | | | | | | Barnett 2019; Crtalic et al. 2015; Dombo et al. 2014; Kellen et al. 2015, Reamer 2013; Reamer 2015; Reamer 2017 |
|  |  |  | | Do not accept clients and former clients as friends | | | | Barsky 2017; Froehlich et al. 2023 |
|  | *Valid/necessary reasons for multiple relations – blurred boundaries – Searching* | Not be motivated by professional’s own gratification | | | | | | Barnett 2019; Gabbard et al. 2011; Jordan et al. 2014; Lannin & Scott 2013 |
|  |  | Motivated by client’s needs | | | | | | Barnett 2019; Barsky 2017; Bratt 2010; Cooper et al. 2019; Frankish et al. 2012; Hartley et al. 2015; Jordan et al. 2014; Kaplan et al. 2011; Kolmes 2012 |
|  | *Postings* | Postings by clients can lead to inadvertent/harmful private disclosure and confidential details (professional is mandated reporter Jordan et al. 2014) | | | | | | Drum; & Littleton 2014; Gabbard et al. 2011; Jordan et al. 2014; Reamer 2015; Reamer 2017; Reamer 2018 |
|  |  | Postings by professionals can lead to inadvertent/harmful private disclosure and confidential details | | | | | | Frankish et al. 2012; Jordan et al. 2014 |
|  |  | Take reasonable steps to prevent patient access to professional’s personal social networking site and do not post personal information on social media | | | | | | Edwards-Stewart et al. 2019; Kellen et al. 2015; Reamer 2018; Smith et al. 2023 |
|  |  | Not share patient’s information on/over professional’s social media (at least de-identify Frankish et al. 2012) | | | | | | Frankish et al. 2012; Gabbard et al. 2011; Lannin & Scott 2013; Kellen et al. 2015; Reamer 2018 |
|  |  | Make sure that content which is posted is correct and up to date | | | | | | Kaslow et al. 2011; Nicholson 2011; Smith et al. 2021; |
|  | *Searching for* | Do not search without client’s knowledge/consent | | | | | | Barsky 2017; Holmes & Reid 2018; Frankish et al. 2012; Jordan et al. 2014; Nicholson 2011; |
|  |  | Some found information may have (strong/detrimental) effects/transference-countertransference (e.g. sexual preference, religion, political views, …) -> how to deal with? | | | | | | Baier 2019; Frankish et al. 2012; Gabbard et al. 2011; Jordan et al. 2014; Kaslow et al. 2011; Nicholson 2011; Reamer 2015; Reamer 2017 |
|  | *Motivation for searching/contacting professional* | Clients being curious about professional | | | | | | Baier 2019; Belkofer et al. 2011; Lannin & Scott 2013 |
|  |  | Having malintent (e.g. stalking, harassment,…) | | | | | | Baier 2019; Bratt 2010; Belkofer et al. 2011 |
|  |  | Clients, especially belonging to marginalised/vulnerable groups may have justified desire to inquire about their professional for safety reasons | | | | | | Kaluzeviciute 2020 |
|  |  | If search is disclosed, be prepared as professional, include in the therapy | | | | | | Dombo et al. 2014 |
|  |  | Clients may never share that they searched for the professional | | | | | | Kolmes 2012 |
|  |  | Clients more likely the initiator | | | | | | Mattison 2018 |
|  | *Motivation for searching client* | Without consent client -> need for sound judgment by professional, be part of treatment (e.g. emergencies like suicide-attempt) | | | | | | Barsky 2017; Belkofer et al. 2011; Cooper et al. 2019; Gabbard et al. 2011; Frankish et al. 2012; Kaplan et al. 2011; Lannin & Scott 2013; Lehavot et al. 2012; Mattison 2018; Reamer 2015; Reamer 2018 |
|  |  | Without consent -> consider as dishonest on part of professional | | | | | | Cooper et al. 2019; Holmes & Reid 2018; Kaslow et al. 2011 |
|  |  | Searches to gather information need to be disclosed | | | | | | Cooper et al. 2019; Holmes & Reid 2018; Frankish et al. 2012; Lannin & Scott 2013; Lehavot et al. 2012; Nicholson 2011; Reamer 2017; Reamer 2018 |
|  |  | Do searches together | | | | | | Holmes & Reid 2018 |
|  | *Availability* | 24/7/365 availability -> plastic boundaries and risk of dependency | | | | | | Belkofer et al. 2011; Cooper et al. 2019; Dombo et al. 2014; Drum; & Littleton 2014; Fantus & Mishna 2013; Holmes & Reid 2018; Reamer 2015; Reamer 2017 |
|  | *Relation boundaries – privacy, confidentiality, trust* | Boundary confusion/crossings/violations -> can impact on privacy, confidentiality of professional and client + impact trust in relationship | | | | | | Baier 2019; Bratt 2010; Drum; & Littleton 2014; Froehlich et al. 2023; Jordan et al. 2014; Kaplan et al. 2011; Kaslow et al. 2011; Kolmes 2012; Lehavot et al. 2012; Kaluzeviciute 2020; Reamer 2013; Reamer 2015; Reamer 2017 |
|  |  | Friending professional -> access to friends list (both client and professional) = breach of confidentiality | | | | | | Crtalic et al. 2015; Jordan et al. 2014; Kaplan et al. 2011 |
| **Privacy, confidentiality and trust** |  | Some issues similar to paper use/face-to-face -> technology differs in magnitude/quality  (Froehlich et al. 2023: respect for privacy/confidentiality across all facets of interaction (online and offline)) | | | | | | Barsky 2017; Drum; & Littleton 2014; Froehlich et al. 2023 |
|  | *Public availability of personal information, self-disclosure* | Personal information available to individuals beyond intended audience, information on internet is permanent, can be pulled out of context (Hartley et al. 2015: Digital footprint) | | | | | | Baier 2019; Barnett 2019; Barsky 2017; Belkofer et al. 2011; Crtalic et al. 2015; Drum & Littleton 2014; Frankish et al. 2012; Gabbard et al. 2011; Hartley et al. 2015; Kolmes 2012; Mattison 2018 |
|  |  | Professionals have responsibility to regulate and restrict media profiles   - Not expect that used technology is safe as standard | | | | | | Baier 2019; Barnett 2019; Cooper et al. 2019; Crtalic et al. 2015; Dombo et al. 2014; Edwards-Stewart et al. 2019; Gabbard et al. 2011; Holmes & Reid 2018; Kaplan et al. 2011; Lannin & Scott 2013; Kellen et al. 2015; Mattison 2018; Reamer 2013; Reamer 2015; Reamer 2017; Reamer 2018 |
|  |  | Strategies to restrict client’s access (not bullet proof)  “Ethical burden on professional to ensure trustworthy encryption” (Dombo et al. 2014; Reamer 2013) | | | | | | Barnett 2019; Cooper et al. 2019; Crtalic et al. 2015; Dombo et al. 2014; Frankish et al. 2012; Froehlich et al. 2023; Gabbard et al. 2011; Holmes & Reid 2018; Kaplan et al. 2011; Lannin & Scott 2013; Reamer 2013; Reamer 2015; Smith et al. 2023 |
|  |  |  | | Creating personal and professional accounts (private accounts can still be found -> can create issues in professional life, see Crtalic et al. 2015) | | | | Barnett 2019; Crtalic et al. 2015; Dombo et al. 2014; Frankish et al. 2012; Froehlich et al. 2023; Gabbard et al. 2011; Hartley et al. 2015; Holmes & Reid 2018; Kaplan et al. 2011; Lannin & Scott 2013; Reamer 2013; Reamer 2015; Smith et al. 2023 |
|  |  |  |  | Use a pseudonym (not fail-safe -> IP Address) | | | | Barnett 2019; Lannin & Scott 2013; Smith et al. 2023 |
|  |  |  |  | Use most restrictive privacy settings | | | | Barnett 2019; Cooper et al. 2019; Frankish et al. 2012; Froehlich et al. 2023; Gabbard et al. 2011; Kaplan et al. 2011; Lannin & Scott 2013; Reamer 2018 |
|  |  |  |  | Be selective about who gets access to information/account | | | | Barnett 2019; Gabbard et al. 2011; Holmes & Reid 2018; Reamer 2018 |
|  |  |  |  | Encryption and firewalls, check regularly | | | | Barsky 2017; Cooper et al. 2019; Crtalic et al. 2015; Froehlich et al. 2023; Jordan et al. 2014; Kaplan et al. 2011; Mattison 2018; Nicholson 2011; Reamer 2013; Reamer 2015; Reamer 2018 |
|  |  | Trying to avoid all self-disclosure = lack of understanding of boundaries and good clinical practice on part of professional  No self-disclosure is impossible | | | | | | Barnett 2019; Barsky 2017; Bratt 2010; Crtalic et al. 2015; Dombo et al. 2014; Fantus & Mishna 2013; Frankish et al. 2012; Lannin & Scott 2013; Nicholson 2011 |
|  |  |  | | Professionals have responsibility to abstain from social media | | | | Baier 2019 |
|  | *Loss of control* | Information always available -> information can already be shared/forwarded by third party, tagging, traced back to source | | | | | | Baier 2019; Barnett 2019; Belkofer et al. 2011; Crtalic et al. 2015; Gabbard et al. 2011; Kaplan et al. 2011; Nicholson 2011; Reamer 2015 |
|  |  | Do self-searches -> see what information about you is available and clean up (some information can put professional in bad daylight, Kaslow et al. 2011) | | | | | | Bratt 2010; Belkofer et al. 2011; Frankish et al. 2012; Dombo et al. 2014; Gabbard et al. 2011; Kaslow et al. 2011; Kolmes 2012; Lannin & Scott 2013 |
|  | *Geotagging* | Problem of geotagging | | | | | | Baier 2019; Nicholson 2011; Smith et al. 2023 |
|  | *Loss of data* | Gaining access by 3^rd^ parties -> e.g. hacking | | | | | | Barsky 2017; Cooper et al. 2019; Crtalic et al. 2015; Dombo et al. 2014; Jordan et al. 2014; Mattison 2018; Reamer 2013; Reamer 2015; Reamer 2018 |
|  |  | Data server (pc, phones, etc.) may be stolen or misplaced, accessed by third parties | | | | | | Barsky 2017; Cooper et al. 2019; Jordan et al. 2014; Nicholson 2011; Reamer 2018 |
|  |  | Data unintentionally send to wrong person | | | | | | Reamer 2018 |
|  |  | Viruses etc. -> loss of data | | | | | | Reamer 2018 |
|  | *Safety technology* | Current (encryption) technology > paper documents -> Some technology is better protected than others | | | | | | Dombo et al. 2014; Edwards-Stewart et al. 2019; Holmes & Reid 2018; Reamer 2013; Reamer 2015; Reamer 2017 |
|  |  | Data breaches mostly done by mishandlings or physical access to device | | | | | | Edwards-Stewart et al. 2019 |
|  | *Different devices* | Use of different devices -> different security -> contain different information | | | | | | Holmes & Reid 2018 |
|  | *Free online services* | Be wary of (free) online services = third parties => client be advised to be careful to share data | | | | | | Barsky 2017; Crtalic et al. 2015; Dombo et al. 2014; Jordan et al. 2014; Kellen et al. 2015; Reamer 2018 |
|  | *Privacy of others* | Ensuring privacy of non-clients (private setting -> family) | | | | | | Drum & Littleton 2014 |
| **Documentation and records** | *Proper documentation* | Need for strict protocols (e.g. about mechanisms to secure back up records and time records will be stored for before being destroyed) to ensure clinically relevant exchanges are properly documented in (online) case records | | | | | | Bratt 2010; Frankish et al. 2012; Holmes & Reid 2018; Nicholson 2011; Reamer 2013; Reamer 2015; Reamer 2017 |
|  | *Disposal/destruction* | Proper disposal and destruction of documents and records | | | | | | Reamer 2015; Reamer 2017 |
|  | *Motivation behind records* | Records necessary for thorough assessment, planning, and delivering services, accountability to clients, agencies, other providers, courts, utilization review organisations, ensure coordination and continuing services, to provide quality supervisions, evaluate services | | | | | | Barsky 2017; Frankish et al. 2012; Reamer 2015; Reamer 2017 |
|  | *Need for documentation* | Documentation of video-counselling, e-mail, text messages, cybertherapy communications, carried out searches | | | | | | Drum; & Littleton 2014; Reamer 2015; Reamer 2017; Reamer 2018 |
|  | *Legislation* | Secure privacy and confidentiality (paper + electronically stored) in line with laws and regulations and be familiar with exceptions | | | | | | Drum; & Littleton 2014; Frankish et al. 2012; Holmes & Reid 2018; Mattison 2018; Nicholson 2011; Reamer 2015; Reamer 2017; Reamer 2018 |
|  |  | Professionals need to know how to respond to subpoenas and court orders to release electronically stored information | | | | | | Reamer 2015; Reamer 2017; Reamer 2018 |
|  | *The deceased* | Management of online data belonging to the deceased | | | | | | Kolmes 2012 |
|  | *Encryption* | Need for good encryption technologies | | | | | | Barsky 2017; Nicholson 2011; Reamer 2013; Reamer 2015; Reamer 2017 |
|  | *Access by professional and client* | Reasonable and appropriate access by clients and colleagues to records and documents | | | | | | Reamer 2015; Reamer 2017 |
| **Competency** | *To promote client’s decision-making/best interests* | Educate client about ethical/boundary/multiple relations/social media issues/privacy, confidentiality issues -> protects professional too   - Client have realistic expectations about treatment relation and process - Client understand how not to use social media in therapy | | | | | | Barnett 2019; Barsky 2017; Belkofer et al. 2011; Cooper et al. 2019; Crtalic et al. 2015; Edwards-Stewart et al. 2019; Fantus & Mishna 2013; Froehlich et al. 2023; Hartley et al. 2015; Holmes & Reid 2018; Kellen et al. 2015; Reamer 2018 |
|  |  | Be sensitive to client’s culture and unique needs (cultural community, linguistic, social economic environment) | | | | | | Barsky 2017; Cooper et al. 2019; Drum; & Littleton 2014; Froehlich et al. 2023; Kolmes 2012; Reamer 2018 |
|  | *Standards* | Need to meet minimal standards of competence (know benefits, risks, how to communicate, how to set privacy settings (Frankish et al. 2012) etc.) | | | | | | Bratt 2010; Belkofer et al. 2011; Cooper et al. 2019; Crtalic et al. 2015; Dombo et al. 2014; Drum & Littleton 2014; Edwards-Stewart et al. 2019; Fantus & Mishna 2013; Frankish et al. 2012; Froehlich et al. 2023; Hartley et al. 2015; Holmes & Reid 2018; Jordan et al. 2014; Kellen et al. 2015; Kolmes 2012; Mattison 2018; Nicholson 2011; Reamer 2013; Reamer 2017; Reamer 2018; Smith et al. 2023 |
|  | *Knowledge* | Review pertinent research & practice literature   - Become familiar with rapidly emerging ethical standards + changing technological landscape - Assess quality of research (best quality = RCT’s), found information | | | | | | Fantus & Mishna 2013; Kaslow et al. 2011; Kellen et al. 2015; Reamer 2013; Reamer 2015; Reamer 2017; Reamer 2018 |
|  |  | Technical mastery, ensure technology works well | | | | | | Cooper et al. 2019; Crtalic et al. 2015; Dombo et al. 2014; Edwards-Stewart et al. 2019; Holmes & Reid 2018; Jordan et al. 2014; Reamer 2015; Reamer 2018; Smith et al. 2023 |
|  | *Training/education* | Seek training/education on using digital technology counselling (e.g. communication challenges such as missing cues) | | | | | | Bratt 2010; Cooper et al. 2019; Dombo et al. 2014; Fantus & Mishna 2013; Gabbard et al. 2011; Holmes & Reid 2018; Jordan et al. 2014; Kaslow et al. 2011; Kolmes 2012; Lannin & Scott 2013; Mattison 2018; Reamer 2015; Reamer 2017; Reamer 2018; Smith et al. 2023 |
|  | *Digital natives – digital immigrants* | Digital natives – digital immigrants might assess online content posted by client differently | | | | | | Lehavot et al. 2012 |
| **Problematic behaviour/Client suitability** | *Assess suitability of client* | Professional must (be able to) assess whether client’s needs can be met using electronic social work services and when necessary to refer client to another professional | | | | | | Barsky 2017; Cooper et al. 2019; Dombo et al. 2014 (see for examples); Drum; & Littleton 2014; Froehlich et al. 2023; Jordan et al. 2014; Mattison 2018; Reamer 2017 |
|  |  |  | Some people with specific difficulties not being served with electronic help | | | | | Reamer 2013 |
|  |  | Telepsychology in the intimacy of one’s home -> create difficulties for clients with histories of disturbed interpersonal boundaries (e.g. client can feel threatened, client can make use of informal language/inappropriate clothing => counterproductive | | | | | | Cooper et al. 2019; Drum & Littleton 2014 |
|  |  | Assessment client’s identity and age -> can be difficult to do online | | | | | | Cooper et al. 2019; Holmes & Reid 2018; Reamer 2017 |
|  |  | Risk of compulsive behaviours regarding the use of technology | | | | | | Reamer 2018 |
| **Consultation, referral, termination/interruption of services** |  | Need to ensure clients are familiar with info they would need to locate and access emergency, counselling, case management, and other supportive services   - Becomes difficult/impossible when: - Professional does not meet the client in person; - professional and client do not live in the same community; - professional does not have a professional relationship with other service providers - Leads to inadequate coordination of services and incomplete/inaccurate clinical assessments | | | | | | Cooper et al. 2019; Crtalic et al. 2015; Dombo et al. 2014; Frankish et al. 2012; Jordan et al. 2014; Kaslow et al. 2011; Kellen et al. 2015; Reamer 2013; Reamer 2018 |
|  | *Emergency* | Need for a third party as emergency contact | | | | | | Cooper et al. 2019; Dombo et al. 2014; Froehlich et al. 2023; Jordan et al. 2014 |
|  | *Termination/interruption of services* | Risk of abandonment -> need to make reasonable arrangements for continuation of services | | | | | | Reamer 2013 |
|  |  | Reasons termination/interruption | | | Clients terminate services abruptly -> disappear, do not responds | | | Reamer 2013 |
|  |  |  |  |  | Professional terminates services -> technical failure, failing to respond in timely manner | | | Crtalic et al. 2015; Reamer 2013 |
|  | *Reasonable alternatives* | If client does not want to rely on technological services | | | | | Make use of face-to-face | Barsky 2017 |
|  |  |  |  |  |  |  | Referral to other professional/agency | Barsky 2017 |
| **Informed consent** | *Protocols and policies* | Need for social media policy for clients and staffers | | | | | | Barsky 2017; Barnett 2019; Belkofer et al. 2011; Cooper et al. 2019; Dombo et al. 2014; Hartley et al. 2015 ; Holmes & Reid 2018; Jordan et al. 2014; Kaslow et al. 2011; Kolmes 2012; Lannin & Scott 2013; Lehavot et al. 2012; Nicholson 2011; Reamer 2013; Reamer 2015; Smith et al. 2023 |
|  |  | Reflecting on and forming protocols about ethical/boundary/multiple relations/conflict of interest/privacy/record and documentation issues/expectations/what to do in emergencies (e.g. friending, not friending, searches etc.)/response time about social media before starting social media use | | | | | | Barnett 2019; Belkofer et al. 2011; Cooper et al. 2019; Crtalic et al. 2015; Dombo et al. 2014; Drum & Littleton 2014; Edwards-Stewart et al. 2019; Fantus & Mishna 2013; Hartley et al. 2015; Holmes & Reid 2018; Jordan et al. 2014; Kaplan et al. 2011; Kaslow et al. 2011; Kellen et al. 2015; Kolmes 2012; Lannin & Scott 2013; Lehavot et al. 2012; Nicholson 2011; Mattison 2018; Reamer 2015; Reamer 2017; Smith et al. 2023 |
|  |  | If protocols/policies are not followed/met revert back to in-person contact | | | | | | Crtalic et al. 2015; Mattison 2018 |
|  | *Informing* | Professional has duty to ensure clients understand nature of electronic services and potential benefits and risks | | | | | | Barsky 2017; Belkofer et al. 2011; Cooper et al. 2019; Edwards-Stewart et al. 2019; Fantus & Mishna 2013; Frankish et al. 2012; Froehlich et al. 2023; Hartley et al. 2015; Kaplan et al. 2011; Kellen et al. 2015; Lannin & Scott 2013; Mattison 2018; Reamer 2013; Reamer 2015; Reamer 2017; Smith et al. 2023 |
|  | *Autonomy* | Ensuring client’s autonomy in informed consent procedure | | | | | | Barsky 2017; Bratt 2010; Hartley et al. 2015; Kaslow et al. 2011; Lehavot et al. 2012; Reamer 2013 |
|  | *Standards of consent* | Client must be mentally capable to give consent (make informed choices, understand relevant facts, circumstances, express wishes)   - Assessing capacities and current circumstances can be difficult when not meeting in person e.g. confirming identity and age | | | | | | Barsky 2017; Cooper et al. 2019; Frankish et al. 2012; Kaplan et al. 2011; Reamer 2013; see Reamer 2015; Reamer 2017 |
|  |  | Online consent forms and procedures must be valid | | | | | | Kaslow et al. 2011; Reamer 2013 |
|  | *Trust* | Informed consent procedure as essential foundation of trust, not seen as risk-management tool, to maintain curiosity -> discuss it regularly (Crtalic et al. 2015;) | | | | | | Crtalic et al. 2015; Kaplan et al. 2011; Kolmes 2012; Mattison 2018 |
|  | *Age* | Difficulty of minors -> state laws differ regarding parental consent | | | | | | Holmes & Reid 2018; Reamer 2013; Reamer 2015; Reamer 2017 |
| **Identity and image** | *Professional’s image* | Professionals have responsibility to ensure online conduct is in accordance with professional and appropriate representations | | | | | | Baier 2019; Barsky 2017; Belkofer et al. 2011; Dombo et al. 2014; Drum; & Littleton 2014; Frankish et al. 2012; Froehlich et al. 2023; Gabbard et al. 2011; Kaluzeviciute 2020; Kellen et al. 2015; Smith et al. 2023 |
|  | *Professional’s identity* | Confirm identity of professional (valid licence, digital certificates) | | | | | | Kaplan et al. 2011; Kaslow et al. 2011; |
|  | *Client’s identity* | Confirm identity of client to whom services are provided at the onset of each contact  Risk of identity theft, fraud | | | | | | Cooper et al. 2019; Crtalic et al. 2015; Holmes & Reid 2018; Kaplan et al. 2011; Mattison 2018; Reamer 2013; Reamer 2017; Reamer 2018 |
|  |  | Client can present himself/herself differently online in an easier way/use of a profile under a pseudonym (Bratt 2010)  Do not take information at face-value | | | | | | Bratt 2010; Cooper et al. 2019; Kaslow et al. 2011; Kaluzeviciute 2020; Kolmes 2012; Lehavot et al. 2012 |
|  |  | Discuss shared password | | | | | | Crtalic et al. 2015; Kaplan et al. 2011 |
|  | *Conflict of interest* | Professional making use of specific site/service -> give impression that professional endorses activities of company/provider | | | | | | Reamer 2013; Reamer 2015; Reamer 2018; Smith et al. 2021 |
| **Others** | *Research evidence* | Very little high quality, compelling research demonstrating evidence of effectiveness | | | | | | Edwards-Stewart et al. 2019; Reamer 2013 |
|  | *Access* | Substantial amount of population not have access to internet/advanced technology | | | | | | Holmes & Reid 2018 |
|  |  | Knowledge of technological devices not be assumed as universal | | | | | | Holmes & Reid 2018 |
|  | *Reviews* | Clients leaving online reviews of professionals | | | | Causing additional anxiety for professional | | Frankish et al. 2012; Kolmes 2012 |
|  |  |  |  |  |  | Feeling gagged by confidentiality requirements and unable to defend themselves against negative reviews and unable to ask positive reviews | | Frankish et al. 2012; Kolmes 2012 |
|  | *Context dependence* | Safety, measures, and (use 0f) technology are context dependent -> context of violent crime =/= context of child care | | | | | | Barsky 2017 |

| **Outliers** | | | |
| --- | --- | --- | --- |
| **Themes** | ***Subthemes*** | **Description** | |
| *Boddy, J. & Dominelli, L. (2017). Social media and social work: The challenges of a new ethical space.* | | | |
| **Macro-level contexts** | Politics – social work | Neoliberal beliefs in market forces and profit making drive political and socio-economic decision + privatized spaces and interpersonal relationships | |
|  |  |  | Commodification of relationships and services -> this logic is taken over by abusers who dehumanize victims |
|  |  |  | Beliefs enable abusive behaviour -> abusers can easily exploit abuse, and control others for profit |
|  |  |  | Free market drives demand for abusive material |
|  |  | Social media prefer profit over safety -> abusers profit from social media’s secrecy => trust in social media is misplaced | |
|  |  | Social media as element of globalisation and technological progress -> made social media ubiquitous and cheaply available everywhere | |
| **Embodiment/disembodiment** | Online relationships | Online relationships can be embodied and contextualized as they inform and are informed by offline reality => blurring physical – digital space | |
|  |  | Bystanders’ reactions + immediate feedback are obscured (disembodiment) | |
|  |  | Dual dynamics: | |
|  |  |  | Simultaneous feelings of connection and disconnection |
|  |  |  | Silencing some voices (e.g. support network) and amplifying other voices (e.g. abusers who know how to control social media) |
| **Single and multiple authorship** | Multi-author set-up of social media | Original author loses control | |
|  |  | Constitutes lack of consent -> number of authors grow => number of audiences grow | |
|  |  | People may lose power of life story (especially children) | |
| **Public and private spaces** | Boundaries | Blurring public - private | |
|  | control | Individuals can modify intended audience -> distributing posted content in own networks; by technical knowledge => eroding privacy in invisible way | |
|  |  | Be aware of challenges related to privacy, security, discretion, respect, data management, accessibility | |
|  |  | Questions of responsibility | |
|  |  | Questions professional boundaries with former clients | |
| **Power and disempowerment** |  | Social media to empower users -> breaking down hierarchical structures | |
|  |  | Social media to disempower users -> lack of control over content, targeted by criminals, marketers, fraudsters, parents losing control over children’s contacts | |
| **Permanence and impermanence** |  | Permanence of evidence of someone’s online engagement | |
|  |  | Impermanence due to speed of communications (= rapid overhaul of information) | |
|  | Power, authorship, consequences | Workers need to be aware of time dimensions in dealing with power, authorship, consequences | |
| **Competence** | Apply values in digital space | Professionals be able to read online spaces and apply their values effectively | |
|  | Critical attitude | Organisations develop comprehensive social work guidelines that cover how to interrogate taken-for-granted assumptions and behave as critically reflective practitioners online | |

| *Steiner, O. (2021). Social Work in the Digital Era: Theoretical, Ethical, and Practical Considerations* | | | |
| --- | --- | --- | --- |
| **Managerialisation of Social Work** | Double-face of digitalisation | Digital technologies reduce human life to numbers, marginalise the meaningful narrative access to a situation by those affected and increasingly allow decisions to be driven by algorithms, rather than by specialised professionals | |
|  |  |  | Automated social exclusion |
|  |  |  | Handing over of responsibility for decision-making |
|  |  |  | Data protection and “function creep” |
|  |  | Digital technologies foster professional conformity with managerialist risk-management systems | |
|  |  | These systems can have upsides, too, though, in: | |
|  |  |  | Communication with clients |
|  |  |  | Counselling |
|  |  |  | Organisation of communities and public relations |
|  |  |  | Support and inclusion of physically and mentally impaired clients |
|  |  | Digital technologies are neither essentially good nor bad, they are formable and fundamentally open | |
| **Core Social Work Concerns** | Social injustice and asymmetric distribution of power | The double-face needs to be assessed from the point of view of social work and its core concerns | |
|  |  | Social work needs to adopt a reflective reaction to digitalisation based on social work values | |
| **Finding a Theoretical Framework of Digitalisation for Social Work** | Inequality and power differences in actor-networks | Using Latour’s actor-network-theory to describe technology as a social phenomenon | |
|  |  |  | All entities (social, natural, or technological) are reciprocally related to each other |
|  |  |  | Networks of actors and technological ‘actants’ perform actions |
|  |  | Adopting Bourdieu’s concepts of field and habitus to reflect structure of inequality and power differences in such networks | |
|  |  |  | How do techno-social networks stabilise and which habitus formations are thereby (re-)actualised? |
|  |  | Castell’s idea of ‘power in digital networks’ and Habermas’ idea of ‘deliberative discourses’ | |
|  |  |  | Can social work participate in public discourses on social media and if yes, how? |
|  |  | Beck’s thesis of reflexive modernisation | |
|  |  |  | Treating questions about digitalisation in practical contexts with regard to discourses on the function of social work in society |
| **Ethics of Responsibility** | Taking responsibility for transforming digital technologies | Necessitated by the highly socially, spatially, and temporally extended effects of digital technology | |
|  |  | For social work, such an ethics raises questions of: | |
|  |  |  | Assuming responsibility for potential problems with digital technologies |
|  |  |  | How digital technologies can be used now and in the future for the empowerment and inclusion of social work clients and the creation of social justice |
|  |  |  | Taking responsibility for developing (formable, fundamentally open) digital technology, especially technically sound software alternatives to managerialist algorithms |

| **Excluded Themes** | | | | |
| --- | --- | --- | --- | --- |
| **Themes** | ***Subthemes*** | **Description** | | **References** |
| **Benefits social media** | *Increased accessibility* | Social media facilitates support groups; community building | | Forehlich et al. 2023 ; Jordan et al. 2014 |
|  |  | People living in urban cities who may not have time/resources to travel to professional | | Barsky 2017 |
|  |  | People who speak a different language | | Barsky 2017 |
|  | *Formal contact -> increased therapeutic value* | Humanizing the therapeutic relationship | | Crtalic et al. 2015; Reamer 2015 |
|  | *Contact* | More informal contact -> cancel/reschedule meetings, brief updates during crisis (real-time updates) | | Reamer 2015; Reamer 2017; Reamer 2018 |
|  |  | Increased contact with colleagues | | Kellen et al. 2015 |
|  | *Effectiveness* | Increased effectiveness of counselling (face-to-face + online counselling) | | Holmes & Reid 2018 |
|  | *Benefit for professional* | Counter therapy fatigue/stress by professional -> increase connection to loved ones etc. | | Kellen et al. 2015 |
|  |  | Expansion of opportunities for information gathering about needs of clients from business perspective | | Froehlich et al. 2023 |
|  |  | Higher visibility of services and own practise | | Crtalic et al. 2015; Kolmes 2012 |
| **Risks social media** |  | Some people with specific difficulties not being served with electronic help | | Reamer 2013 |
|  |  | Loss of reasonable expectation of a turnaround time for responses   - May lead to misunderstanding & conflict | | Cooper et al. 2019; Reamer 2013 |
|  |  | Risk of violating state laws | | Reamer 2013 |
|  |  | Risk of encountering identity fraud | | Reamer 2013 |
|  |  | Digital technology is disquieting | | Reamer 2015 |
|  |  | Digital technology is abhorrent and unethical | | Baier 2019; Reamer 2015 |
|  |  | Diluting the meaning of the therapeutic relation and alliance | | Reamer 2015 |
|  |  | In-person contact contains deep connection which is necessary for practice | | Reamer 2015 |
|  |  | Difficulty in setting clear boundaries in part due to ambiguity of temporal limits of online interactions | | Reamer 2015 |
|  |  | Challenges protecting and managing client’s privacy and confidentiality, access by unauthorized 3^rd^ parties | | Mattison 2018; Reamer 2013; Reamer 2015; Reamer 2018 |
|  |  | Development rigorous standards of care/evidence of effectiveness lagging behind development of technology used in behavioural professions | | Dombo et al. 2014; Edwards-Stewart et al. 2019; Reamer 2018 |
| **Self-disclosure, friending, relations, limits, and boundaries** | *Real – virtual therapeutic relationship* | No control over remote location | | Cooper et al. 2019 |
|  | *Personal relationships over social media* | Avoid providing services to people with whom professional has personal relationship with via social media | | Reamer 2017 |
|  | *Negative Consequences multiple relations – blurred boundaries – self-disclosures – Friending* | Not all clients will disclose their online actions -> Professional will lack knowledge and so control over self-disclosure | | Kaluzeviciute 2020; Kaslow et al. 2011; Kolmes 2012 |
|  | *Valid/necessary reasons for multiple relations – blurred boundaries – Searching* | Traverse boundaries in a manner that is welcomed by client | | Barnett 2019; Frankish et al. 2012; Lannin & Scott 2013 |
|  |  | Consistent with professional’s theoretical orientation | | Barnett 2019; Kolmes 2012 |
|  |  | Respectful of relevant diverse factors | | Barnett 2019; Bratt 2010; Frankish et al. 2012 |
|  |  | Comports with professional practice standard | | Barnett 2019 |
|  |  | Make sure that content which is posted is correct and up to date | | Kaslow et al. 2011; Nicholson 2011; Smith et al. 2023 |
|  | *Searching for* | Not disclosing a search done denies client and professional opportunity to process the disclosure within therapeutic environment | | Baier 2019; Nicholson 2011; Reamer 2018 |
|  |  | Ethical and legal ramifications | | Belkofer et al. 2011; Bratt 2010; Gabbard et al. 2011 |
|  | *Small scale ethics* | Digital world = small community | | Frankish et al. 2012; Lannin & Scott 2013 ; Nicholson 2011 |
| **Privacy, confidentiality and trust** | *Public availability of personal information, self-disclosure* | - Support client (Edwards-Stewart et al. 2019) | | Edwards-Stewart et al. 2019 |
|  | *Privacy legislation* | Compliance with Health Information Technology for Economic and Clinical Health (HITECH) | | Edwards-Stewart et al. 2019 |
|  |  | Compliance with HIPAA and other regulations, standards, statuses | | Cooper et al. 2019; Crtalic et al. 2015; Dombo et al. 2014; Edwards-Stewart et al. 2019; Froehlich et al. 2023; Holmes & Reid 2018; Kaplan et al. 2011; Kellen et al. 2015; Lannin & Scott 2013; Reamer 2013; Reamer 2015; Reamer 2018 |
|  |  | Client-generated data on client’s mobile device not covered by legal standards | | Edwards-Stewart et al. 2019 |
|  | *Loss of control* | virtual chance encounters, e.g. professional and client member of same online group | | Barnett 2019; Reamer 2018 |
|  | *Video-counselling* | Much less confidential than traditional settings | 3^rd^ parties that sit in a sessions (e.g. family member, friend) outside camera without professional knowledge or consent | Reamer 2015; Reamer 2017 |
|  |  |  | Encryption not always ensured | Reamer 2015 |
|  | *Online group counselling* | Confidentiality agreements when doing online group treatment | | Reamer 2015; Reamer 2017 |
| **Documentation and records** | *Proper documentation* | Of decision-making steps | | Reamer 2015 |
|  | *Motivation behind records* | Who has access? | | Barsky 2017 |
|  | *Legislation* | Comply with laws and regulations concerning protection of electronically-stored information | | Holmes & Reid 2018; Reamer 2015; Reamer 2017 |
| **Competency** | *Knowledge* | Also technical knowledge when professional does not use social media, chances are client does use it | | Belkofer et al. 2011; Lannin & Scott 2013 |
|  |  | Communicating with public over social media -> professionals take reasonable steps to ensure accuracy and validity of disseminated information | | Frankish et al. 2012; Nicholson 2011; Reamer 2018 |
|  | *Being familiar with multiple relationships* | Professional needs to become familiar with nature of multiple relationships and ethical decision-making models | | Kolmes 2012 (has a lot of examples); Lannin & Scott 2013 |
| **Problematic behaviour/Client suitability** | *Professional’s problematic behaviour* | Professionals not create unscrupulous websites or online posts designed to exploit people financially (e.g. encouraging to buy products) | | Reamer 2018 |
|  |  | Avoid engaging in harassment/cyberbullying, doxing, threats, trolling, cyber mob attacks, hate speech | | Smith et al. 2023 |
|  | *Emergency* | Need for a third party as emergency contact | | Cooper et al. 2019; Dombo et al. 2014; Jordan et al. 2014 |
| **Informed consent** | *Standards of consent* | Avoid coercion, undue influence, pressure | | Barsky 2017; Reamer 2013 |
|  | *Information* | Using social media page/account -> include disclaimer about purposes and limitations of page/account | | Froehlich et al. 2023 |
|  | *Context-dependent* | Case-by-case assessments -> e.g. victim domestic violence -> important to know how information will be searched | | Barsky 2017 |
| **Identity and image** | *Professional’s image* | Client will engage in solipsistic introjection -> creating fantasy of always available professional based on online content -> can cause frustration/confusion when expectation is not met | | Bratt 2010; Drum; & Littleton 2014; Kaluzeviciute 2020 |
| **Others** | *Critique on small-scale ethics (Lannin & Scott 2013)* | Risks perceiving an inaccurate element of inevitability to online self-disclosure | | Baier 2019 |
|  | *Research evidence* | Practice involving social media should be evidence-based | | Kaslow et al. 2011 |
|  | *Licensing* | Services across state lines (USA) -> different licensing laws, regulations, policies  Have licences in both states | | Barsky 2017; Cooper et al. 2019; Holmes & Reid 2018; Kaplan et al. 2011; Reamer 2013; Reamer 2015; Reamer 2018 |
|  |  | Most interjurisdictional and international telepsychology practice is illegal (under US law) | | Cooper et al. 2019; Reamer 2013 |
|  |  | Obtain legal consultations when necessary | | Holmes & Reid 2018; Reamer 2015 |
|  | *Collegial relationships* | Ensure to treat colleagues with respect when posting comments online | | Lannin & Scott 2013; Reamer 2015; Reamer 2017; Reamer 2018 |
|  |  | Avoid cyberbullying and collegial harassment | | Lannin & Scott 2013; Reamer 2015; Reamer 2017; Reamer 2018 |
|  |  | Avoid derogatory and defamatory postings | | Gabbard et al. 2011; Lannin & Scott 2013; Reamer 2015; Reamer 2018 |
|  |  | Respect colleagues’ privacy | | Reamer 2015; Reamer 2018 |
|  |  | Respect colleagues’ online work products | | Reamer 2015; Reamer 2017; Reamer 2018 |
|  |  | Respond appropriately to colleagues’ unethical behaviour; not using safeguards, report them | | Reamer 2015; Reamer 2017; Reamer 2018 |
|  |  | Consult colleagues as self-monitoring strategy | | Bratt 2010; Lannin & Scott 2013; Reamer 2015; Reamer 2018; Smith et al. 2023 |
|  |  | Obtain appropriate supervision (if available) | | Lannin & Scott 2013; Reamer 2015; Reamer 2018 |
|  |  | Avoid gratuitous and unwarranted searches about colleagues for unprofessional purposes | | Reamer 2017 |
|  |  | Take reasonable steps to correct/remove inaccurate/offensive information they have posted/transmitted (about colleagues) | | Barsky 2017; Reamer 2017; Reamer 2018; Smith et al. 2021 |
|  |  | Newly developed relationships on business-related social media should remain limited to professional relationships | | Kaslow et al. 2011; Smith et al. 2021 |
|  | *Beneficence* | May demand immediate intervention by professional | | Lehavot et al. 2012 |
|  |  | Intervention may also cause harm -> undermining self-expression + diminishing trust in clinician | | Lehavot et al. 2012 |
|  | *Reviews* | Professionals boosting their online reviews | | Frankish et al. 2012; Kolmes 2012 |
